# Strategic disruption of cancer’s powerhouse: precise nanomedicine targeting of mitochondrial metabolism

**DOI:** 10.1186/s12951-024-02585-3

**Published:** 2024-06-08

**Authors:** Pei Lin, Ye Lu, Jiarong Zheng, Yunfan Lin, Xinyuan Zhao, Li Cui

**Affiliations:** 1 Stomatological Hospital, School of Stomatology, Southern Medical University, Guangzhou, 510280 Guangdong China; 2 Department of Dentistry, The First Affiliated Hospital, Sun Yat-Sen University, Guangzhou, 510080 China; 3 School of Dentistry, University of California, Los Angeles, Los Angeles, CA 90095 USA

**Keywords:** Mitochondria, Metabolism, Nanomedicine, Cancer therapy

## Abstract

Mitochondria occupy a central role in the biology of most eukaryotic cells, functioning as the hub of oxidative metabolism where sugars, fats, and amino acids are ultimately oxidized to release energy. This crucial function fuels a variety of cellular activities. Disruption in mitochondrial metabolism is a common feature in many diseases, including cancer, neurodegenerative conditions and cardiovascular diseases. Targeting tumor cell mitochondrial metabolism with multifunctional nanosystems emerges as a promising strategy for enhancing therapeutic efficacy against cancer. This review comprehensively outlines the pathways of mitochondrial metabolism, emphasizing their critical roles in cellular energy production and metabolic regulation. The associations between aberrant mitochondrial metabolism and the initiation and progression of cancer are highlighted, illustrating how these metabolic disruptions contribute to oncogenesis and tumor sustainability. More importantly, innovative strategies employing nanomedicines to precisely target mitochondrial metabolic pathways in cancer therapy are fully explored. Furthermore, key challenges and future directions in this field are identified and discussed. Collectively, this review provides a comprehensive understanding of the current state and future potential of nanomedicine in targeting mitochondrial metabolism, offering insights for developing more effective cancer therapies.

## Background

Mitochondria, the cell’s bioenergetic hubs, comprise four key components: the outer mitochondrial membrane (OMM), the intermembrane space (IMS), the inner mitochondrial membrane (IMM), and the mitochondrial matrix (MM) [1]. They are central to cellular energy metabolism, playing a crucial role in the oxidation of various metabolic substrates, including carbohydrates, fats, and proteins. These substrates are converted into intermediates like acetyl coenzyme A (acetyl-CoA), which then enters the mitochondria for further processing in the tricarboxylic acid (TCA) cycle, underscoring the organelles’ critical role in energy production. Additionally, mitochondria are pivotal in regulating key cellular processes like redox balance, Ca^2+^ levels, apoptosis initiation, and biomacromolecule balance, thereby influencing cell survival and death [2].

Mitochondrial metabolic pathways predominantly encompass the TCA cycle, oxidative phosphorylation (OXPHOS), fatty acid oxidation (FAO) and glutamine (Gln) metabolism. These pathways are integral to the breakdown of biomacromolecules and the generation of energy [3]. Importantly, mitochondria serve as a primary source of reactive oxygen species (ROS), stemming from their involvement in various core metabolic processes. The production of ROS can disrupt the redox network, leading to the oxidation of lipids and proteins, mutations in mitochondrial DNA (mtDNA), and the induction of oxidative stress. These effects are implicated in the initiation and progression of a range of systemic diseases, including cancer, neurodegenerative disorders, diabetes, obesity, ischemic heart disease, hyperthyroidism, and phenylketonuria [4–6].

Tumor cells, to meet the demands of rapid proliferation, often activate or alter mitochondrial metabolic pathways to secure additional energy. This metabolic reprogramming, encompassing phenomena such as the increased glycolysis, Gln dependency, and lipid metabolism dysregulation, not only supports the growth of cancer cells but also aids in evading immune surveillance and resistance to conventional therapies [7]. Consequently, therapeutic strategies targeting mitochondrial metabolic pathways are increasingly recognized in cancer treatment. This includes the development of novel drugs targeting mitochondrial functions to modulate ROS production, alter the metabolic state of tumor cells, or induce apoptosis. In-depth understanding of mitochondrial metabolic pathways also contributes to unraveling mechanisms of drug resistance in cancer, providing insights for more effective treatment approaches [8]. As the complex interplay between mitochondrial metabolism and tumor development becomes further elucidated, targeting mitochondrial metabolism is anticipated to bring new breakthroughs in cancer therapy.

In this review, we critically analyze mitochondrial metabolism’s role in cellular function and its association with cancer progression, providing several novel insights. The review thoroughly discusses mitochondrial metabolic pathways and their aberrations in cancer cells. Importantly, it systematically reviews the latest advancements in nanomedicine, focusing on targeting mitochondrial metabolism to improve cancer treatment outcomes. It underscores the potential of these nanotechnologies in reprogramming cancer cell metabolism. Finally, we identify future challenges and prospects in employing strategies targeting mitochondrial metabolism for cancer treatment.

## Mitochondrial metabolic pathways

### TCA cycle

The TCA cycle, also known as the Krebs or citric acid cycle, occurs within the mitochondrial matrix and is central to energy production, biosynthesis of biomolecules, and redox balance. Carbon from glucose, fatty acids, or Gln, in the form of acetyl-CoA, initiates this cycle. This leads to the generation of electron carriers like nicotinamide adenine dinucleotide (NADH) and flavin adenine dinucleotide (FADH2), which then transfer electrons to the electron transport chain (ETC) in the IMM. This process results in the production of CO_2_ and ATP [9]. While acetyl-CoA is the primary entry point of the TCA cycle, alternative pathways exist through intermediates such as alpha-ketoglutarate (α-KG), oxaloacetate, fumarate, and succinyl-CoA, derived from amino acid metabolism. Importantly, TCA cycle intermediates also function as signaling molecules, impacting chromatin modification, DNA methylation, hypoxic response, and immune functions, thus playing critical roles in cellular regulation [10, 11]. Specifically, succinate, when released into the cytoplasm, activates various pathways, including HIF-1α, influencing inflammation, hypoxia, and metabolic signaling [12, 13]. Additionally, succinate regulates cellular adaptability by inhibiting α-KG-dependent dioxygenases, highlighting its broad regulatory capabilities [14].

### OXPHOS

OXPHOS is pivotal in eukaryotic cellular metabolism and energy production, primarily operating through the ETC in the IMM [15]. This chain comprises four protein complexes: complex I (CI, NADH-ubiquinone oxidoreductase), complex II (CII, succinate dehydrogenase), dimeric complex III_2 (CIII_2, cytochrome bc1 oxidoreductase), and complex IV (CIV, cytochrome c oxidase), along with two mobile electron carriers, ubiquinone and cytochrome c2 [16]. The ETC facilitates electron transfer from donors like NADH and FADH2, produced in the TCA cycle, to oxygen, the final electron acceptor. This electron flow generates a proton gradient across the IMM, leading to a negative membrane potential (ΔΨm) of -160 to -180 mV, which is essential for ATP synthesis by F0F1-ATP synthase [15, 17]. In this process, complexes I and III generate excessive ROS, including oxygen radicals and hydrogen peroxide (H_2_O_2_), which can damage key cellular components such as lipids, nucleic acids, and proteins. Specifically, ROS can cause oxidative damage to purine and pyrimidine bases, DNA strand breaks, and disrupt DNA repair processes, leading to gene mutations and genetic instability that potentially promote cellular transformation into cancer [18]. Additionally, ROS induce oxidative modifications of redox-sensitive amino acid residues in regulatory proteins, including cysteine oxidation. These modifications can impair the function of key regulatory proteins such as kinases (MAPK and PI3K/Akt), transcription factors (NRF2, AP-1, NF-κB, STAT3, and p53), as well as components of the ubiquitin/proteasome system and the autophagy/lysosomal protein degradation system, disrupting cellular homeostasis and contributing to carcinogenesis [19]. Furthermore, ROS target lipids, leading to peroxidation and the production of genotoxic molecules such as malondialdehyde, acrolein, and 4-hydroxynonenal. This lipid peroxidation can alter membrane structure, disrupt membrane permeability, and interrupt immune system recognition, cumulatively promoting the occurrence and progression of cancer [20].

In addition to cancer, ROS are critical contributors to diseases associated with mitochondrial dysfunction [21]. For instance, mitochondrial dysfunction driven by the Lrrk2G2019S mutation elevates ROS production, initiating the recruitment of gasdermin D to mitochondrial membranes and activating necroptosis via the RIPK1/RIPK3/MLKL pathways. This process is linked to severe immune responses in Lrrk2G2019S mice infected with *Mycobacterium tuberculosis*, underscoring the critical role of mitochondrial ROS in driving immunopathology and highlighting potential targeted interventions [22]. Additionally, increased NOX4 in astrocytes intensifies Alzheimer’s disease pathology by impairing mitochondrial metabolism, boosting mitochondrial ROS production, and triggering lipid peroxidation. This cascade promotes ferroptosis, underscoring NOX4’s pivotal role in oxidative stress-related neurodegeneration [23]. Moreover, noise exposure induces oxidative stress in mice, leading to mitochondrial dysfunction in the cochlea and resultant damage to ribbon synapses. This disruption contributes significantly to noise-induced hearing loss by reducing mitochondrial membrane potential and elevating markers of oxidative damage, highlighting the critical role of ROS in auditory dysfunction [24].

Importantly, the ETC is also vital in generating and sustaining the mitochondrial membrane potential. This stability is essential not only for ATP generation but also for cellular survival. A compromised mitochondrial membrane potential can precipitate a rapid decline in cellular energy supply, and the resultant release of cytochrome c into the cytoplasm triggers the intrinsic apoptotic pathway [12, 25].

### FAO

FAO, or β-oxidation, represents the primary pathway for fatty acid degradation, offering a substantial source of cellular energy. This process generates over twice the ATP yield compared to carbohydrates or proteins. Fatty acids serve as the predominant energy source during limited glucose availability, such as post-absorption and fasting states, and in conditions with ample glucose, fueling vital organs like the heart, skeletal muscles, and kidneys [26].

In the cytoplasm, fatty acids undergo activation by CoA synthetase to form acyl-CoA esters. These esters are pivotal for various metabolic pathways, including lipid synthesis and FAO [27]. FAO itself is a cyclic catabolic process, executed by a trifunctional enzyme complex in the mitochondrial matrix. The carnitine palmitoyltransferase (CPT) system, comprising two acyltransferases (CPT1 and CPT2) and the carnitine acylcarnitine translocase, plays an essential role in transporting acyl-CoA into the mitochondria for β-oxidation [28]. Within the mitochondrial matrix, acyl-CoA is systematically shortened by two-carbon units in a four-step enzymatic cycle, producing acetyl-CoA for the TCA cycle and ketogenesis, alongside FADH2 and NADH for ATP production via the respiratory chain. The physicochemical properties of acyl-CoA alter as it is progressively shortened, necessitating distinct β-oxidation mechanisms with specialized enzymes for complete degradation of varying chain lengths [26, 27]. Dysregulation in FAO, either through abnormal activation or inhibition, can disrupt normal energy metabolism, leading to oxidative stress and inflammatory responses. This imbalance potentially triggers acute or chronic inflammatory diseases [12, 29].

### Gln and glutamate (glu) metabolism

Gln, one of the most abundant amino acids in human plasma, is a key nutrient for cell proliferation and the main form of nitrogen transport between organs. Although considered a non-essential amino acid, Gln is an essential substrate for the biosynthesis of other amino acids, glutathione (GSH), hexosamines, and nucleotides, and can also generate energy through the TCA cycle metabolism [30]. Gln is first converted to Glu by glutaminase (GLS), and then Glu can either enter the TCA cycle by being transformed into α-KG via glutamate dehydrogenase (GDH) or cellular transaminases—this process also reduces NAD^+^ to NADH. Additionally, Glu supports the synthesis of fatty acids and non-essential amino acids such as aspartate, alanine, arginine, and proline, and is essential along with Gln for producing GSH, a crucial intracellular antioxidant [31, 32].

## Abnormal mitochondrial metabolism and cancer

### ROS imbalance and cancer

Mitochondria, a major source of ROS through the ETC, play a pivotal role in cell survival and apoptosis [33]. Although electron transfer in the ETC is efficient, an estimated 0.2-2% of electrons leak and react with O_2_ to form superoxide [34, 35]. Superoxide is rapidly converted into H_2_O_2_ by superoxide dismutase (SOD). H_2_O_2_ functions as a key intracellular messenger of ROS or transforms into more reactive hydroxyl radicals (OH^−^) in the presence of metal ions. Additionally, superoxide can react with nitric oxide (NO) to form reactive nitrogen species (RNS) [36–38].

The imbalance in ROS is a defining trait of cancer cell phenotypes. ROS, which can induce carcinogenesis by causing mutations or promoting cancer cell survival, proliferation, metastasis, and genetic instability, drive tumor progression [37]. For instance, oxidative stress can lead to chronic inflammation, cancer progression, and tumor drug resistance by activating key transcription factors, including HIF-1α, β-catenin/Wnt, and NF-κB [20]. Notably, ROS serve as a critical stimulant in the signaling pathways of tumor angiogenesis. For example, in mononuclear cells (MNCs), elevated ROS levels stabilize HIF-1α, which in turn increases the secretion of vascular endothelial growth factor and macrophage migration inhibitory factor. These factors are crucial in regulating the chemoresistance of MNCs [39]. Furthermore, recent studies indicate that ROS-mediated modulation of the activity of redox-sensitive deubiquitinating enzymes, such as USP7 and A20, is linked to the progression of leukemia, liver cancer, and metastatic cancers [40]. Additionally, ROS activate signaling pathways such as MAPK and PI3K-AKT, promoting cell proliferation [41]. Furthermore, elevated mitochondrial ROS levels can induce “mitohormesis,” a self-regulatory mitochondrial response to mild stress, activating mTOR complex 1-mediated signaling pathways that promote tumor cell proliferation and resistance to apoptosis [37]. However, an excess of ROS can trigger oxidative stress and cell death, thereby exhibiting anti-tumor effects. The “ROS set point theory” highlights the complex interplay between tumor cells and ROS levels, suggesting that cancer cells modulate their antioxidant systems and decrease mitochondrial OXPHOS to maintain ROS within a range that promotes oncogenesis [42].

### Mitochondrial-driven metabolic reprogramming in cancer

In cancer development, tumor cells undergo metabolic reprogramming to support uncontrolled growth and metastatic progression [43]. This process, characterized by enhanced aerobic glycolysis, also involves significant changes in nitrogen metabolism. The utilization of Gln nitrogen is redirected from fueling the TCA cycle to nucleotide biosynthesis [44]. In healthy cells, glutamine’s nitrogen primarily supports the TCA cycle and basic cellular functions. In contrast, cancer cells exploit this nitrogen for nucleotide synthesis, essential for DNA and RNA production and rapid proliferation. This shift, facilitated by key enzymes such as glutaminase and phosphoribosyl pyrophosphate amidotransferase (PPAT), enhances the high proliferation rates typical of aggressive tumors. Targeting this pathway, particularly by modulating the PPAT/GLS1 ratio, has shown efficacy in inhibiting tumor growth across various cancers, notably neuroendocrine tumors like small cell lung cancer. Additionally, the regulation of glycinamide ribonucleotide formyltransferase trifunctional protein (GART) by Vestigial-like family member 3 (VGLL3) in lung and breast cancer cells further illustrates the crucial role of glutamine-derived nucleotides in sustaining rapid tumor growth. Inhibition of GART or VGLL3 reduces cell proliferation, while supplementation with inosine monophosphate, a downstream metabolite of GART, can restore this growth, underscoring the essential nature of this metabolic pathway in cancer progression [45].

**Fig. 1:**
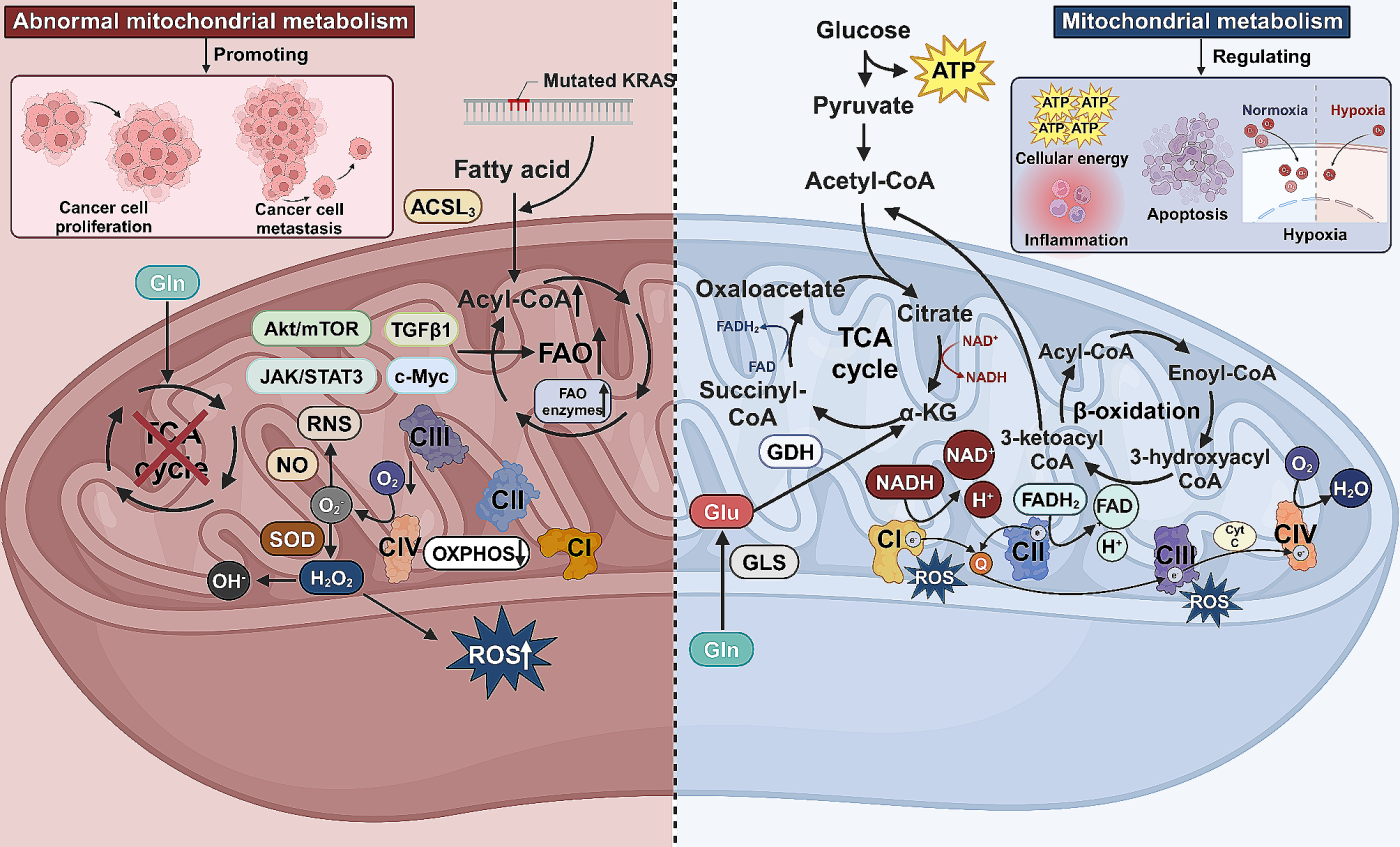
Mitochondrial metabolism and its key role in cellular energy dynamics and cancer development. In cellular energy dynamics, key mitochondrial metabolic pathways such as the TCA cycle, OXPHOS, FAO, and Gln and Glu metabolism play pivotal roles. The TCA cycle, essential for energy production, initiates with acetyl-CoA, leading to the generation of electron carriers NADH and FADH2. TCA intermediates also significantly influence cellular processes, including inflammation and hypoxia. OXPHOS, occurring along the ETC in the mitochondrial inner membrane, creates a proton gradient essential for ATP synthesis and concurrently generates ROS. FAO, the primary pathway for fatty acid degradation, takes place in the mitochondrial matrix, cyclically breaking down fatty acids into acetyl-CoA and electron carriers, thereby facilitating energy production. Glu, crucial for cell proliferation and nitrogen transport, is converted into glutamate and subsequently into α-KG, integrating into the TCA cycle and supporting various biosynthetic pathways. Mitochondria are central in ROS generation through the ETC, a process vital for cell survival and apoptosis. However, a small proportion of electrons in the ETC leads to the formation of superoxide, which is converted into H_2_O_2_ and RNS, implicated in the mechanisms of carcinogenesis. Cancer cells undergo metabolic reprogramming characterized by increased reliance on aerobic glycolysis and shifts in nitrogen metabolism, diverting from traditional TCA cycle operations towards nucleotide biosynthesis. Additionally, alterations in FAO have been identified as critical factors in cancer development, affecting cancer cell proliferation and survival

Importantly, abnormalities in FAO are increasingly recognized as key contributors to cancer development, influencing cell proliferation, survival, stemness, drug resistance, and metastasis [46]. Elevated FAO activity has been identified in several cancer types, including KRAS mutated lung cancer, triple-negative breast cancer, and gliomas [47–49]. Mutated KRAS in tumors has been shown to regulate intracellular fatty acid metabolism via ACSL3, promoting the conversion of fatty acids to acyl-CoA esters. This conversion increases the substrates available for lipid synthesis and β-oxidation, which are crucial for tumor development. Proteins involved in the FAO pathway, such as CPT1A, CPT1B, CPT1C, CPT2, the carnitine transporter CT2, and ACSL3, are often overexpressed in various cancers [50]. High expression levels of these FAO enzymes are closely related to poor prognosis in patients [51, 52]. For example, CPT1A is highly expressed in nasopharyngeal carcinoma (NPC) cells and tissue samples, where it plays a key role in modulating the malignant phenotypes of NPC, including proliferation, anchorage-independent growth, and tumor formation in mouse xenograft models. CPT1A has been identified as a critical factor responsible for the aberrant activation of FAO in NPC cells. Its upregulation promotes ATP generation through OXPHOS in the mitochondria, as well as facilitates the production of key precursors for nucleic acid biosynthesis [53]. The upregulation of FAO may be driven by tumor-promoting molecules like Akt/mTOR, TGFβ1, c-Myc, and JAK/STAT3, which contribute to the metabolic reprogramming of tumor cells [54–57]. Notably, mitochondrial lipid metabolism plays a critical role in ferroptosis, a regulated form of cell death triggered by lipid peroxidation and reliant on iron [58]. For instance, inhibition of BRD4 can suppress mitochondrial fatty acid oxidative metabolism, reducing the accumulation of lipid peroxides and thereby protecting cells from ferroptosis, making BRD4 a potential regulatory link between lipid metabolism and ferroptosis [59]. This discovery provides new insights into targeting BRD4 to modulate mitochondrial lipid metabolism for cancer treatment **(**Fig. 1**)**.

## Overview of nanomedicine in biomedical applications

**Fig. 2:**
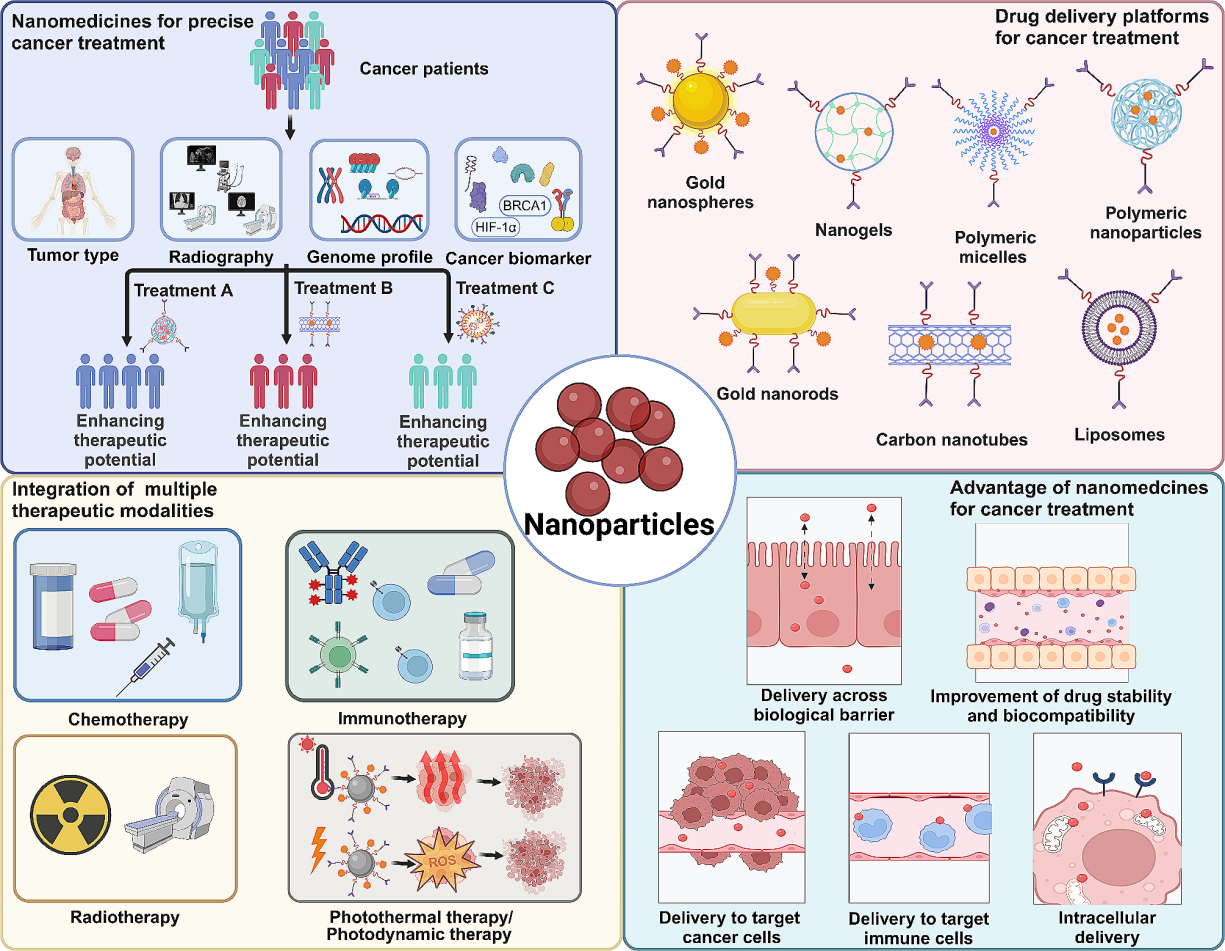
Enhancing cancer therapy efficacy and precision with nanoparticles. Nanoparticles are emerging as personalized treatment vectors, augmenting the efficacy of tumor therapies. Diverse nanoparticle forms, including gold nanospheres, nanogels, polymeric micelles, gold nanorods, carbon nanotubes, and liposomes, have been employed in cancer treatments as advanced drug delivery systems. Notably, the inherent multifunctionality of nanoparticles synergizes various therapeutic modalities such as chemotherapy, immunotherapy, radiotherapy, and phototherapy, thereby enhancing treatment effectiveness. Additionally, nanoparticles possess the unique ability to traverse biological barriers, enabling targeted delivery to cancer cells and specific intracellular sub-organelles while enhancing drug stability and biocompatibility

The advancement of nanobiomaterials, particularly in the realm of medicine, has been significant in recent years. This progression facilitates intricate targeting strategies and multifunctionality, effectively reducing the adverse side effects typically associated with drug overdosage [60, 61]. Diverse nanomaterials, including those derived from organic, inorganic, lipid, and polysaccharide compounds, as well as synthetic polymers, have been critical in the development and enhancement of novel therapeutic modalities **(**Fig. 2**)** [62]. For instance, nanomaterial-based drug delivery systems have been used to enhance the efficacy of various therapies such as photothermal therapy (PTT), photodynamic therapy (PDT), radiotherapy, chemotherapy, and immunotherapy, showing potential for long-term anti-cancer effects [63–66]. Additionally, lipid nanoparticles have been effectively used as carriers for nucleic acid-based drugs [67]. These advancements demonstrate substantial promise in both the diagnosis and treatment of a wide range of diseases [62].

### Drug delivery system

A primary impact of nanotechnology has been its transformative role in the development of drug delivery systems. This advancement has led to marked improvements in the biodistribution and pharmacokinetics of pharmaceutical agents [68]. Drugs formulated with nanomaterials exploit their unique structural properties to enhance therapeutic efficacy, including controlled release of the drug, safeguarding of molecular integrity, facilitation of biological barrier penetration, and precise delivery to targeted sites [69, 70]. Furthermore, drug delivery systems utilizing nanocarriers have shown improvements in drug stability and biocompatibility, alongside an extension in the circulation lifespan of these therapeutic molecules within the bloodstream. Such advancements significantly enhance drug accumulation within targeted tissues, thereby improving therapeutic outcomes [71].

Importantly, nanomedicines enhance therapeutic strategies by co-delivering drugs and imaging agents, enabling the visualization and quantification of drug delivery to target sites. This approach assists in predicting therapeutic efficacy and refining dosing regimens [72]. For instance, The Sur@T7-AIE-Gd nanoparticle, synthesized for liver cancer therapy, combines dual-mode imaging and delivers survivin siRNA with low toxicity and high efficacy. It effectively targets liver tumors, inhibiting growth and impacting cell DNA, making it a promising tool for image-guided siRNA therapy in hepatocellular carcinoma [73].

Different drug delivery systems, including inorganic nanoparticles, inorganic-organic hybrid nanosystems, lipid nanoparticles, extracellular vesicles, and protein-based nanomaterials, have been utilized to enhance therapeutic efficacy [74, 75]. For example, biodegradable mesoporous silica nanoparticles (bMSN) have been developed as a theranostic tool for guided PDT and personalized cancer immunotherapy. With an efficient delivery of neoantigen peptides, adjuvants, and photosensitizers, bMSN facilitates PET imaging and PDT, enhancing local and systemic antitumor responses [76]. Recent advances in MOF-based drug delivery have led to the development of multi-biomimetic nanocarriers that integrate ferroptosis induction and PDT, enhancing cancer treatment efficacy. These nanocarriers, cloaked with cancer cell membranes for targeted delivery, induce ROS upon cellular uptake, promoting cell death and tumor suppression [77]. Additionally, LNPs have been optimized for targeted mRNA delivery to specific organs, notably the lungs, thereby enhancing the efficacy of RNA-based therapies. A library screening approach revealed that N-series LNPs, which feature an amide bond, efficiently transport mRNA to pulmonary cells. This targeted delivery has been successfully utilized to reduce tumor burden in a preclinical lung disease model, demonstrating the potential of mRNA LNPs in treating respiratory diseases [78, 79]. Moreover, CBSA/siS100A4@Exosome nanoparticles have been developed to target lung pre-metastatic niches in triple-negative breast cancer, showing a notable preference for lung tissue over liposomal formulations. These 200 nm-sized nanoparticles effectively deliver siRNA, protect it from degradation, and exhibit enhanced biocompatibility and gene-silencing effects, significantly reducing the growth of malignant cells and potentially curbing postoperative metastasis [80]. Importantly, as protein-based nanomaterials, nanoerythrocytes membranes coated with chitosan and poly (lactide-co-glycolic acid) demonstrate improved pharmacokinetics and biodistribution, ensuring prolonged drug release and increased uptake by the liver, thus boosting the therapeutic efficacy against liver cancer [81].

### Synergistic integration of therapeutic modalities in a single nanoplatform

The concept of a single nanoplatform integrating multiple therapeutic modalities presents a promising avenue in modern medical treatments. The use of nanomaterials to amalgamate drugs with differing action mechanisms, or to facilitate combined therapies such as drug delivery, radio/chemotherapy, photodynamic, and thermal therapy, has shown synergistic effects [82]. For instance, Xu et al. created the Di-DAS-VER NPs, a nanosystem combining a ROS-sensitive dasatinib (DAS) prodrug with verteporfin (VER), a photosensitizer. This nanosystem, targeting choroidal neovascularization in wet age-related macular degeneration, releases DAS and generates ROS through VER upon red light exposure, effectively inhibiting neovascularization with minimal systemic toxicity [83]. In the context of cancer therapy, reliance on single-agent chemotherapy often leads to multi-drug resistance, diminishing its efficacy and causing adverse effects, such as gastrointestinal toxicity, liver damage, bone marrow suppression, and immunosuppression, due to its deleterious impact on normal tissues [84]. In addition, the intricate tumor microenvironment further complicates this scenario by impeding drug penetration and diffusion, thereby limiting chemotherapy’s effectiveness [85, 86]. Combined therapy based on nanomaterial-based drug delivery systems thus emerges as a promising strategy. Liu et al. developed a multifunctional core-shell nanosystem featuring a zinc oxide (ZnO) core and a polydopamine (PDA) shell, integrating chemo-, gene-, and photothermal therapies. The system combines doxorubicin (Dox) and DNAzyme with the PDA shell, releasing Zn^2+^ from the ZnO core to activate DNAzyme for gene silencing. This approach shows enhanced anti-tumor effects and reduced side effects of Dox [87].

## Precisely targeting mitochondrial metabolism with advanced nanomedicine

Mitochondria, characterized by their double-membrane structure, are essential organelles housing mtDNA, various ions, and proteins. Their architecture is further complicated by the presence of cardiolipin [88]. The dynamic nature of mitochondria, evident in their continuous mitotic and fusion-fission activities, presents challenges in developing targeted therapeutics [42].

Innovative approaches have been proposed to address these challenges, notably through the integration of mitochondria-targeting molecules within nanoparticle frameworks. For instance, a mitochondria-targeted diketopyrrolopyrrole photosensitizer has been developed, enabling photodynamic and photothermal anti-cancer therapies by targeting mitochondria for heat and singlet oxygen generation [89]. It precisely targets mitochondrial by incorporating imidazole groups, which utilize the cationic nature of imidazole to interact with the mitochondrial membrane’s inner-negative and outer-positive potential. Additionally, the commonly used ligand, triphenylphosphonium (TPP), characterized by a deuterated cationic charge and containing three phenyl rings, exhibits both lipophilic and hydrophilic properties, enabling TPP-modified molecules to easily accumulate in the mitochondrial matrix through the lipid bilayer. In another advancement, a mitochondria-targeting conjugate, CTPP-CSOSA, combines lipophilic cationic (4-carboxybutyl) triphenylphosphonium bromide (CTPP) with a glycolipid-like compound (CSOSA). This conjugate responds to mitochondrial alkaline pH, enhancing reactive ROS production and inducing mitochondrial permeabilization, which leads to increased apoptosis in tumor cells [90]. Notably, in addition to using ligands, such as CTPP, TPP, and the heptamethine cyanine dye IR68, to target mitochondria, various materials have been explored for the construction of mitochondrial-targeting nanocarriers. These materials include liposomes; polymeric nanocarriers; protein nanoparticles; and inorganic nanoparticles [91–94]. Lipid-based delivery technologies leverage the ability of liposomes to fuse with mitochondrial membranes, thereby directly releasing drugs or genetic materials inside cells. For instance, the treatment of osteoarthritis has utilized fusion mitochondrial capsules (FMCs) synthesized with neutral lipids, cationic lipids, aromatic lipids, and three types of liposomes (FMC0, FMC1, and FMC2) [95]. Lipophilic cation-based delivery systems exploit their cationic properties to penetrate cellular lipid layers and accumulate within mitochondria, driven by potential gradients [96]. These strategies not only enhance the precision of mitochondrial targeting but also reduce drug exposure to non-specific tissues, thereby aiming to improve therapeutic outcomes and minimize side effects. Additionally, emerging nanomaterials such as MOFs, carbon nanoparticles, and black phosphorus nano-materials are being investigated as potential mitochondrial delivery platforms [97–99].

### Enhanced ROS by targeting mitochondria

#### Metal-based nanoparticles

Metal-based nanoparticles are increasingly utilized in cancer therapy due to their ability to enhance oxidative stress-mediated cancer cell death. They achieve this either by boosting the production of endogenous ROS within mitochondria or by forming and delivering exogenous ROS directly within cells. This approach leverages the unique properties of these nanoparticles to disrupt cellular oxidative balance, resulting in oxidative stress and selectively targeting cancer cells while minimizing harm to normal cells. For instance, zinc peroxide nanoparticles (ZnO_2_ NPs) are developed for cancer treatment by generating both endogenous and exogenous ROS. These nanoparticles release H_2_O_2_ and Zn^2+^, enhancing mitochondrial ROS production and effectively killing cancer cells. Additionally, manganese-doped ZnO_2_ NPs function as MRI contrast agents, providing diagnostic capabilities [100]. Likewise, zero-valent-iron nanoparticles (ZVI-NPs) effectively suppress lung cancer progression by disrupting mitochondrial function and increasing oxidative stress in cancer cells [101]. Interestingly, a Bi_2_S_3_-based nanoneedle, doped with iron (FeBS), simplifies mitochondria-targeted therapy by integrating photodynamic and photothermal effects. Its uniquely designed lipophilic and positively charged surface specifically targets mitochondria, while iron doping enhances ROS production, improving therapy effectiveness [102]. Moreover, gold nanoparticles (AuNPs), enhanced with polymer and folate, accumulate in mitochondria, leading to oxidative stress and apoptosis. Notably, AuNPs disrupt glycolysis, reducing key enzymes through a c-Myc-dependent pathway, causing energy deprivation and inhibiting tumor growth, with minimal impact on non-tumor cells [103].

#### Inorganic nonmetallic materials

Inorganic nonmetallic nanodrugs have also demonstrated outstanding performance and broad application potential in treating tumors by inducing oxidative stress through targeting mitochondria. For example, iron-oxide magnetic nanoparticles conjugated with Dox effectively target mitochondrial dysfunction in breast cancer cells, inducing oxidative stress that leads to DNA damage, lipid peroxidation, and mitochondrial potential loss. This mitochondrial disruption halts the cell cycle and reduces cell migration, enhancing Dox delivery and increasing cancer cell mortality while potentially lowering toxicity to healthy cells, highlighting their promise in anticancer therapy [104]. Similarly, superparamagnetic iron oxide nanoparticles (SPIONs) demonstrate selective cytotoxicity in vitro against mitochondria isolated from oral cancer. Exposure to SPIONs increases ROS production, disrupts mitochondrial membrane potential, triggers cytochrome c release, and induces mitochondrial swelling. Furthermore, SPIONs decrease succinate dehydrogenase activity in cancerous mitochondria, suggesting their potential as therapeutic agents for oral cancer without significantly affecting non-cancerous cells [105]. Notably, CsI(Na)@MgO nanoparticles, paired with 5-aminolevulinic acid, present a streamlined radiodynamic therapy strategy enhancing tumor suppression. This combination targets mitochondria in cancer cells, increasing ROS production upon X-ray exposure and synergizing with DNA-targeted irradiation to intensify mitochondrial, DNA, and lipid damage [106]. Furthermore, ZnO nanoparticles effectively inhibit proliferation and induce apoptosis in human multiple myeloma cells, primarily through mitochondria-mediated pathways. Exposure to ZnO nanoparticles increases ROS production and decreases ATP levels, enhancing cytochrome C, APAF-1, caspase-9, and caspase-3 expression. This suggests a potent role for ZnO nanoparticles in triggering mitochondrial apoptosis, with minimal cytotoxic effects on peripheral blood mononuclear cells, highlighting their potential as therapeutic agents against multiple myeloma [107].

#### Organic nanoparticles

Organic nanocarriers offer significant advantages for drug delivery, including enhanced cellular penetration due to their small size and surface modification capabilities. These carriers exhibit high target specificity, improved drug stability, and controlled release mechanisms responsive to specific stimuli like pH or temperature. Additionally, their biocompatibility and low toxicity are crucial for reducing side effects and improving patient tolerance [108]. For instance, red-emissive carbon dots (RCDs) have been developed for PDT, with tunable ROS generation suitable for both aerobic and hypoxic conditions. These RCDs can produce both type I and type II ROS, owing to their specific core sizes and surface states. Their mitochondrial targeting ability enables them to activate cell death via mitochondrial pathways [109]. Interestingly, the nanocarrier HAL/3MA@X-MP, combining hexyl 5-aminolevulinate hydrochloride (HAL) and 3-methyladenine (3MA) within tumor cell-derived microparticles (X-MP), targets tumor cells. HAL induces sonosensitizer accumulation in mitochondria for effective ROS generation and mitochondrial damage, while 3MA inhibits mitophagy and downregulates PD-L1, enhancing immunogenic cell death and impeding immune checkpoint recognition [110]. Moreover, a mitochondria-targeted drug nanocarrier, prepared through host-guest interactions between α-cyclodextrin and polyethylene glycol (PEG), effectively combines a NO donor and a cinnamaldehyde prodrug for cancer treatment. This approach enhances oxidative stress by depleting GSH and generating peroxynitrite in mitochondria, leading to effective apoptosis in cancer cells and showing significant antitumor activity in hepatoma models [111].

#### Organic/inorganic hybrid nanoparticles

Organic/inorganic hybrid nanomaterials demonstrate significant advantages in drug delivery. By combining the stability and unique optical properties of inorganic components with the biocompatibility of organic components, these materials facilitate efficient drug loading and precise control over drug release, enhancing accumulation at targeted sites [112]. One commonly employed strategy is to integrate metals or metal compounds with other organic components to form composite nanomedicines. For instance, using bio-synthesized gold nanoclusters (Au NCs) paired with mitochondria-targeted aptamer-Pyro conjugates (ApPCs), this approach enhances uptake and mitochondrial targeting within cancer cells. When irradiated, it produces high levels of ROS, effectively killing cancer cells [113]. Similarly, mdGC is a nanoformulation created by modifying gold nanorods with carbon-based nanomaterials. It triggers tumor cell apoptosis by increasing ROS and affecting mitochondrial function, leading to a high tumor targeting rate and significant tumor growth reduction, without needing external laser sources [114]. In addition, MitoCAT-g combines carbon-dot-supported gold with triphenylphosphine and cinnamaldehyde, targeting mitochondria to deplete GSH and amplify oxidative stress. This leads to apoptosis in cancer cells and effective tumor growth inhibition in hepatocellular carcinoma models [115]. Beyond the gold nanomaterials, various other metals and metal-containing compounds are also being actively explored and utilized. For example, a novel nanocomposite with hyaluronic acid-modified calcium and copper peroxides effectively targets tumors. It releases calcium, copper, and H_2_O_2_ in tumor environments, triggering hydroxyl radical production and increasing oxidative stress, leading to mitochondrial damage [116]. Notably, a nanoplatform, incorporating molybdenum disulfide nanoflakes and hyperbranched polyglycerol, effectively targets and delivers PDT agents to both mitochondria and endoplasmic reticulum, significantly enhancing cellular uptake in resistant cells. It effectively triggers ROS generation, leading to substantial tumor reduction by inducing both ER stress and mitochondrial dysfunction, thereby reversing drug resistance in cancer therapy [117]. Moreover, BPQD-PEG-TPP combines phosphorus quantum dots (BPQDs) with a heterobifunctional PEG and a TPP group for effective mitochondrial targeting. This nanoplatform excels in its photothermal properties, enhancing ROS production and demonstrating significant photothermal cytotoxicity against cancer cells [118]. Additionally, the MB@Bu@MnO2@Alb nanoparticles deliver butformin, a mitochondrial-targeted analog, and methylene blue to reverse hypoxia and disrupt the PD-1/PD-L1 axis, thereby enhancing the generation of ROS and promoting immunogenic cell death [119]. Combining bioactive compounds with inorganic nanomaterials enables efficient therapeutic delivery. For example, the electrical pulse-mediated turmeric silver nanoparticle therapy combines turmeric’s bioactive compounds with physical delivery methods, significantly reducing triple-negative breast cancer (TNBC) cell viability and having minimal impact on non-cancerous cells. Proteomic analysis shows changes in key signaling pathways and metabolism, shifting TNBC cells towards mitochondrial processes and increasing ROS [120].

MOFs, a novel class of highly porous materials, are exemplary of organic/inorganic hybrid nanomaterials. They are constructed from metal ions or clusters coordinated with organic linkers through coordination bonds. The uniqueness of MOFs lies in their highly ordered porous structure, allowing for precise control over pore size and shape. These materials are notable for their exceptionally high surface area and customizable porosity, making them highly versatile in various applications, including gas storage, separation, catalysis, drug delivery, and sensing [121]. For instance, BSArGO@ZIF-8 NSs is a multifunctional nanoplatform designed for cancer therapy, using MOF nanoparticles on a graphene oxide surface. It disrupts cancer cells by overloading them with Zn^2+^, increasing ROS, and inducing apoptosis through mitochondrial events and autophagy pathways. Its photothermal capability further enhances tumor suppression, demonstrating potential in combined cancer therapy strategies [122]. Similarly, a MOF (PCN-224) encapsulated in a ROS-responsive amphiphilic copolymer and loaded with carbon monoxide releasing molecule (CORM-401) targets mitochondria for enhanced PDT in cancer treatment. This mitochondria-focused approach, activated by near-infrared light, induces apoptosis and ferroptosis, significantly improving antitumor efficacy [123].

#### Other novel nanobiomaterials

**Fig. 3:**
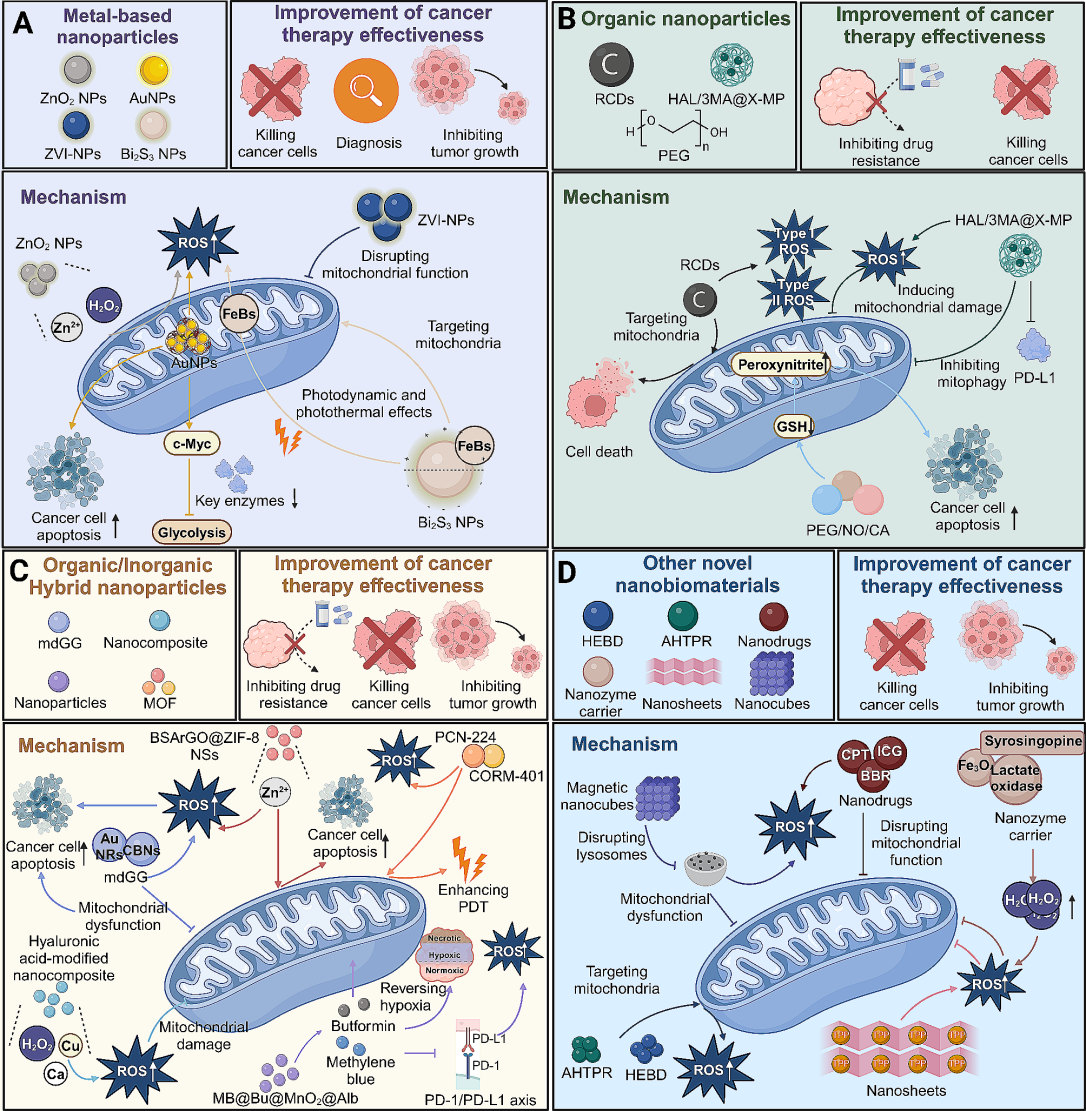
Nanoparticles targeting mitochondria to enhance ROS generation and improve cancer treatment. **(A)** Metal-based nanoparticles, including ZnO_2_ NPs, AuNPs, ZVI-NP, and Bi_2_S_3_-based nanoneedles, specifically target mitochondria. They disrupt mitochondrial function and generate ROS, thereby mediating apoptosis in cancer cells, inhibiting tumor growth, and facilitating early diagnosis. **(B)** Organic nanoparticles, such as RCDs, HAL/3MA@X-MP and PEG, induce effective ROS generation and mitochondrial damage. This leads to the activation of cell death pathways and mediates apoptosis in cancer cells. **(C)** Organic/inorganic hybrid nanoparticles, including mdGC, MB@Bu@MnO2@Alb, and MOF nanoparticles, increase ROS production and affect mitochondrial function. This contributes to apoptosis in cancer cells, inhibition of drug resistance, and suppression of tumor growth. **(D)** Other novel nanoparticles, such as HEBD, AHTPR, and nanosheets enhanced with TPP, as well as Fe_3_O_4_ nanozyme carriers, disrupt mitochondrial function, enhance ROS production, and accelerate cancer cell apoptosis, effectively inhibiting tumor growth

Innovative nanomaterials have been engineered to specifically target mitochondrial metabolism, thereby modulating ROS levels in cancer cells **(**Fig. 3**)**. A mechanical-thermal induction therapy utilizes magnetic nanocubes and alternating magnetic fields to increase ROS levels in cancer cells, causing cell death. This innovative approach disrupts lysosomes, leading to mitochondrial dysfunction and a rise in ROS [124]. Interestingly, a one-pot nanoconstruction (HEBD) is synthesized through chemical reactions among epigallocatechin gallate, buthionine sulfoximine, and formaldehyde in water, with subsequent Dox adsorption. This nanoconstruction targets mitochondria to disrupt electron transport and increase ROS production, enhancing Dox’s chemodynamic therapy, improving anti-tumor efficacy, prolonging survival in tumor models, and reducing cardiotoxicity and metastasis risks [125]. Likewise, nanodrugs combining camptothecin (CPT) and berberine (BBR) with indocyanine green (ICG) disassemble under stimuli, releasing drugs rapidly into cancer cells. Their dual approach of chemotherapy and PTT disrupts mitochondrial function, enhances ROS, and accelerates cancer cell apoptosis, effectively inhibiting tumor growth [126]. Moreover, a hollow Fe_3_O_4_ nanozyme carrier, co-loaded with lactate oxidase and syrosingopine, inhibits lactate efflux, enhancing intracellular acidification and H_2_O_2_ production, leading to significant ROS generation. This process disrupts mitochondrial function and energy supply in tumor cells. Additionally, it restructures the tumor immune environment, boosting anti-cancer immunity and synergizing chemodynamic, immunological, and starvation therapies for effective cancer treatment [127]. Notably, a novel nanoplatform (AIBI@H-mMnO2-TPP@PDA-RGD, AHTPR) combines low-temperature PTT with thermodynamic therapy for effective cancer treatment. It specifically targets mitochondria in cancer cells, releasing radicals to induce cell death at temperatures below 45 °C. Importantly, AHTPR effectively depletes GSH, enhancing therapy effectiveness against hypoxic tumors [128]. Interestingly, a novel cancer treatment approach uses 2D WS_2_ nanosheets, enhanced with TPP for mitochondria targeting, and loaded with the glycolysis inhibitor FX11. These nanosheets, under ultrasound, generate ROS to target and disrupt cancer cell mitochondria and energy metabolism [129] **(**Table 1**)**.

**Table 1:** Nanoparticles targeting mitochondria to enhance ROS generation and improve cancer treatment

Type	Composition	Mechanisms	Effects	Outcomes	Ref.
ZnO_2_NP	ZnO_2_	Inhibit ETC	↑ROS, ↑oxidative stress	Kill cancer cells	[100]
ZVI NP	Zero-valent-iron	Cause mitochondria dysfunction	↑ROS, ↑oxidative stress ↑ferroptotic death	Kill cancer cells, reprogram TME	[101]
FeBS NP	Bi_2_S_3_-based nanoneedle, Fe ion	Produce extra ROS	↑ROS, ↑oxidative stress ↑photothermal effect	Inhibit tumor growth, enhance PDT / PTT efficacy	[102]
AuNP	Polymer, folate, Au	Impair mitochondrial structure / function, inhibit GLUT1 / HK2	↑ROS, ↑oxidative stress ↑mitochondrial apoptosis	Inhibit tumor growth	[103]
RCDs	Carbon dots	Produce type I/II ROS	↑ROS, ↑mitochondrial apoptosis	Kill cancer cells	[109]
HAL/3MA @X-MP	HAL, 3MA, tumor cell-derived microparticle	Produce ROS, inhibit mitophagy, downregulate PD-L1	↑ROS, ↑oxidative stress ↑mitochondrial damage, ↑ICD	Kill cancer cells	[110]
T-NPCA/NO	NO donor, cinnamaldehyde, α-CD, PEG	Produce ROS, deplete GSH, cause mitochondrial dysfunction	↑ROS, ↑oxidative stress↑ mitochondrial apoptosis	Enhance antitumor ability of oxidative therapy	[111]
Au NCs -Ap PCs	Au NCs, ApPCs	Produce ROS, disrupt mitochondrial membrane	↑ROS, ↑oxidative stress	Kill cancer cells, inhibit tumor growth, enhance PDT efficacy	[113]
mdGC	Au NRs, CBNs	Increase intracellular ROS, reduce mitochondrial membrane potential	↑ROS, ↑oxidative stress↑ Mitochondrial apoptosis	Kill cancer cells, inhibit tumor growth	[114]
MitoCAT-g	CAT-g, TPP, cinnamaldehyde	Produce ROS, deplete GSH	↑ROS, ↑oxidative stress, ↑oxidative stress, apoptosis	Kill cancer cells, inhibit tumor growth	[115]
CaO_2_/CuO_2_@HA NC	HA, calcium peroxides, copper peroxides	Produce ROS, Ca^2+^ overload, mitochondrial dysfunction	↑ROS, ↑oxidative stress	Promote tumor calcification and necrosis	[116]
Cy7.5-TG@GPM	MoS_2_, hPG, organelle-targeting PDT agents	Produce ROS, mitochondrial dysfunction, cytochrome C release	↑ROS, ↑oxidative stress, ↑cancer cell apoptosis	Kill cancer cells, enhance PDT efficacy, reverse MDR	[117]
BPQD-PEG-TPP	BP quantum dots, heterobifunctional PEG, TPP	Produce ROS	↑ROS, ↑oxidative stress ↑photothermal effect	Enhance PPT efficacy	[118]
MB@Bu@MnO2@Alb	Manganese dioxide albumin, MB, Bu	Disrupt OXPHOS, inhibit PD-L1 and PD-1, reverse hypoxia	↑ROS, ↑ICD, reverse TIME	Kill cancer cells, inhibit tumor growth, enhance PDT efficacy	[119]
Tur NP	Tur NP	Enhance pyruvate metabolism, TCA cycle and OXPHOS	↑ROS	Kill cancer cells, enhance ECT efficacy	[120]
BSArGO @ZIF-8	ZIF-8 NPs, GO, bovine serum albumin	Zn^2+^overload, initiate bim, upregulate PUMA/NOXA, downregulate Bid/p53AIP1	↑ROS, ↑autophagy, ↑Mitochondrial apoptosis	Kill cancer cells, enhance IIT efficacy	[122]
PCN-224	Zirconium, 4-caboxyphenyl porphyrin, CORM-401, HA, TPP	Produce ROS, triggers intracellular release of CO	↑ROS, ↑apoptosis, ↑ferroptosis	Kill cancer cells, enhance PDT efficacy	[123]
Magnetic nanocubes	Magnetic nanocubes	Increase ROS, disrupt lysosomes, mitochondrial dysfunction	↑ROS, ↑oxidative stress	Kill cancer cells, eradicate glioma and breast cancer	[124]
HEBD	EGCG, BSO, formaldehyde, Dox	Disrupt mitochondrial ETC, inhibit GSH biosynthesis	↑ROS, ↑mitochondrial apoptosis, ↓hypoxia	Kill cancer cells, enhance CDT efficacy	[125]
CPT-ss-BBR/ICG	CPT, BBR, ICG	Produce ROS, disrupt mitochondria membrane potential	↑ROS, ↑cancer cell apoptosis	Inhibit tumor growth, enhance therapeutic efficacy	[126]
Syr/LOD @HFN	Hollow Fe_3_O_4_ catalytic nanozyme carrier, LOD, Syr	Inhibit lactate efflux, produce ROS, damage mitochondria	↑ROS, ↑oxidative stress, remodel TME	Enhance therapeutic efficacy	[127]
AHTPR	RGD, PDA, TPP, H-mMnO_2_	Produce ROS, deplete GSH,	↑ROS, ↑mitochondrial apoptosis	Inhibit tumor growth, enhance efficacy of LPTT and TDT	[128]
FX11@TPEG-WS2	FX11, TPP, poly PEG, WS2 nanosheet	Produce ROS, inhibit glycolysis	↑ROS, ↓ATP	Inhibit tumor growth, enhance chemotherapy efficacy	[129]

### Disrupted mitochondrial energy metabolism

#### Targeting mitochondrial OXPHOS

**Fig. 4:**
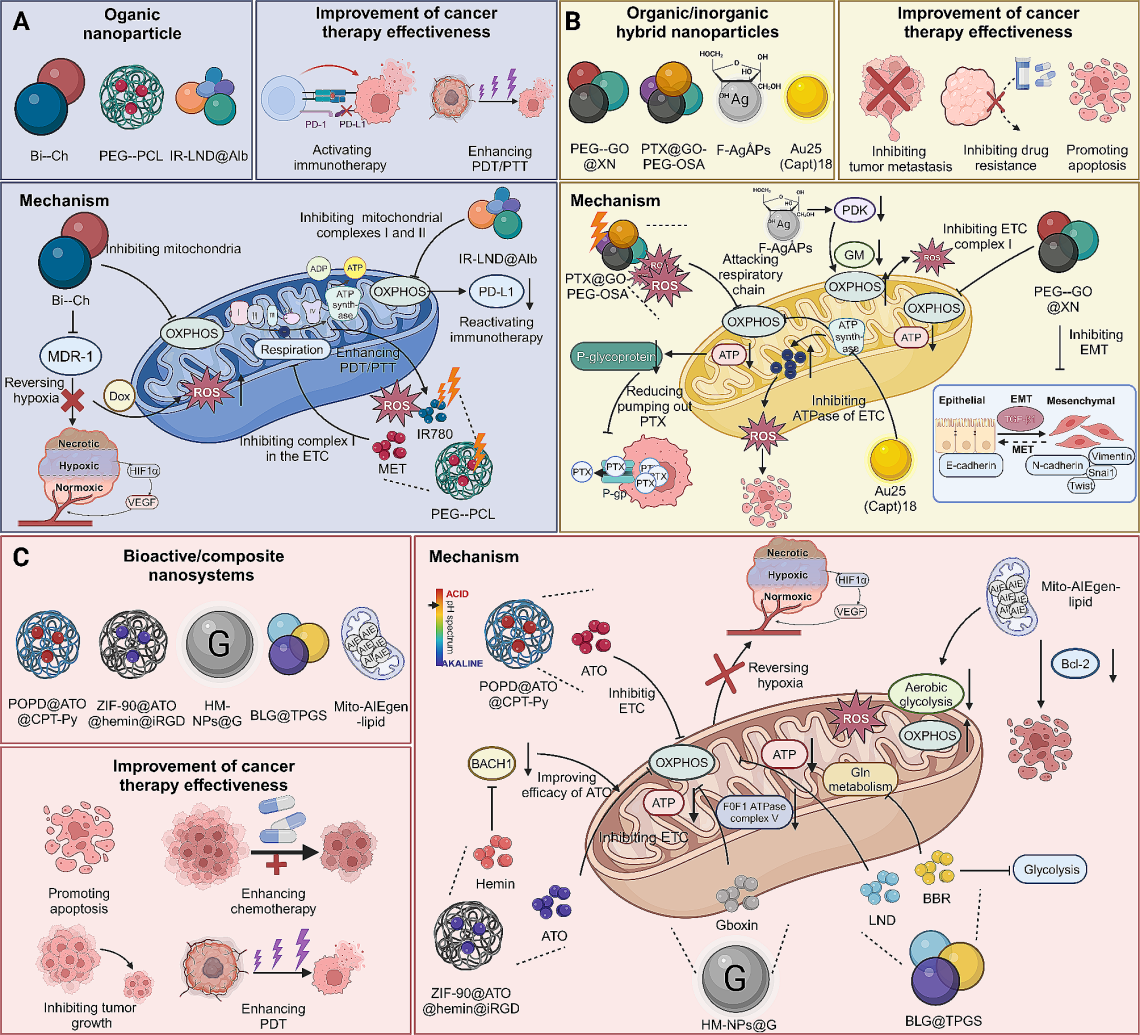
Nanoparticles targeting mitochondria inhibit OXPHOS to enhance cancer treatment. **(A)** Organic nanoparticles, such as Bi-Ch, PEG-PCL, and IR-LND@Alb, inhibit OXPHOS and reverse hypoxia, thus enhancing the effectiveness of oxygen-sensitive therapies, including chemotherapy and PDT. **(B)** Organic/Inorganic hybrid nanoparticles, including PEG-GO@XN, PTX@GO-PEG-OSA, F-AgAps, and Au25(Capt)18, impede mitochondrial respiratory chain enzymes, resulting in increased ROS and reduced ATP production, leading to cell apoptosis and impairing mechanisms of cell migration and invasion while augmenting chemotherapy efficacy. **(C)** Other novel nanoparticles, such as BLG@TPGS, POPD@ATO@CPT-Py, ZIF-90@ATO@hemin@IRGD, and HM-NPs@G, also inhibit OXPHOS. Contrastingly, Mito-AIEgen-lipid shifts cellular metabolism towards OXPHOS, thereby inhibiting growth, triggering cell apoptosis, and enhancing the efficiency of PDT

Similar to targeting mitochondria to enhance ROS in cancer cells, various types of nanoparticles have been developed to target mitochondrial OXPHOS, thereby suppressing tumor growth and progression **(**Fig. 4**)**. As regards to organic nanoparticles, biguanide-modified chitosan targets mitochondrial OXPHOS in cancer cells, reducing tumor hypoxia and multidrug resistance protein 1 expression. This enhances the effectiveness of oxygen-sensitive therapies like chemotherapy, increasing Dox accumulation and cytotoxicity in tumor cells [130]. Similarly, PEG-PCL liposomes co-delivering IR780 and metformin (Met) also represent a novel approach in targeting tumor hypoxia. Met inhibits mitochondrial complex I, reducing respiration and overcoming hypoxia. This facilitates effective combined photodynamic and photothermic therapy using IR780. The treatment also enables near-infrared/photoacoustic imaging, offering a novel solution to hypoxia-induced resistance in cancer therapies [131]. Notably, IR-LND@Alb nanoparticles are created by conjugating the mitochondrial-targeted dye IR-68 with lonidamine (LND), an agent that inhibits mitochondrial complexes I and II, and then self-assembling with albumin (Alb). This approach effectively reduces PD-L1 expression, surpassing the anti-tumor efficacy of conventional anti-PD-L1 monoclonal antibodies. Additionally, IR-LND serves as a promising PDT drug with self-oxygen and self-PD-L1 regulation capabilities. This strategy offers a new avenue for mitochondrial-targeted immunotherapy and enhanced PDT in cancer treatments [91].

In the context of organic/inorganic hybrid nanoparticles, the PEG-GO@XN nanocomposite, integrating prenylated chalcone xanthohumol with graphene oxide, selectively targets OXPHOS in metastatic breast cancer cells. It reduces ATP production, disrupts cell migration and invasion mechanisms, and inhibits metastasis to the lung in mice. PEG-GO@XN also prevents epithelial-mesenchymal transition in cancer cells, indicating its potential as a targeted treatment for metastatic breast cancer [132]. Similarly, PTX@GO-PEG-OSA, a drug delivery system, enhances paclitaxel’s efficacy against gastric cancer by integrating with modified graphene oxide nanosheets. It releases drugs in response to pH and temperature changes and generates ROS under near-infrared irradiation, targeting mitochondrial respiratory chain enzymes [133]. Moreover, fructose-coated Ångstrom-scale silver particles (F-AgÅPs) demonstrate significant anti-osteosarcoma effects, outperforming cisplatin in tumor growth inhibition and metastasis prevention, with minimal toxicity. These particles induce apoptosis in osteosarcoma cells by shifting glucose metabolism from glycolysis to mitochondrial oxidation [134]. In addition, Au25(Capt)18 (CNC) exhibits significant cytotoxicity in cancer cells by disrupting mitochondrial functions, specifically targeting OXPHOS and ATP synthase, leading to increased ROS and apoptosis. This activity occurs independently of external light excitation, highlighting the crucial role of ligand shells in nanocluster applications [135].

Interestingly, the composite nanomaterials have also demonstrated promising efficacy in targeting mitochondrial OXPHOS for treating cancer. For instance, the supramolecular nanoplatform, comprising an amphiphilic polymer, phototherapeutic agent Cy7-CN, atovaquone (ATO), and CPT, targets hypoxic tumor tissues for PDT. ATO inhibits mitochondrial OXPHOS, improving oxygen availability in tumors and enhancing PDT efficiency aiding oxygen use in PDT. This approach, combining enhanced PDT and PTT with chemotherapy, shows promising tumor inhibition results [136]. Similarly, a ZIF-90 based nanocarrier, loaded with ATO and hemin and modified with iRGD, reprograms mitochondrial metabolism in breast cancer treatment. Hemin degrades BACH1, boosting ATO’s inhibition of mitochondrial respiration. The nanocarrier’s ATP-responsive, dual-targeting approach effectively concentrates ATO in mitochondria, enhancing tumor suppression with minimal side effects [137]. Gboxin, aslo known as an inhibitor of OXPHOS, specifically targets complex V (F0F1 ATP synthase). HM-NPs@G, coated with a cancer cell-mitochondria hybrid membrane, improves biocompatibility, pharmacokinetics, blood-brain barrier (BBB) permeability, and dual targeting. This nanomedicine includes an ROS-responsive polymer for controlled Gboxin release, enhancing blood circulation and tumor targeting, effectively inhibiting glioblastoma (GBM) growth in mice with minimal side effects [138]. Moreover, the BLG@TPGS nanocomposite is a synergistic nano-therapeutic modality prepared by doping with multiple energy inhibitors — BBR and LND — along with the chemotherapeutic agent gambogic acid. This approach effectively cuts off mitochondrial respiration, glycolysis, and Gln metabolism, significantly reducing tumor proliferation and migration [139]. In addition, some novel bioactive nanosystems have been designed to achieve anti-tumor effects by modulating the mitochondrial metabolic pathways in cancer cells. A mitochondrial-targeting aggregation-induced emission luminogen (AIEgen), DCPy, is combined with living mitochondria to form a bioactive nanohybrid for deep-seated cancer treatment. This nanohybrid generates ROS under microwave irradiation, inducing apoptosis in cancer cells and reprogramming their metabolism from glycolysis to OXPHOS, thereby further enhancing the efficiency of microwave dynamic therapy [140]. Likewise, a novel cancer treatment strategy combines an aggregation-induced emission photosensitizer with bioactive mitochondria (Mito-AIEgen-lipid) to enhance PDT efficiency. This engineered living system shifts cell metabolism towards OXPHOS, inhibiting growth and triggering apoptosis [141].

#### Targeting ATP production

**Fig. 5:**
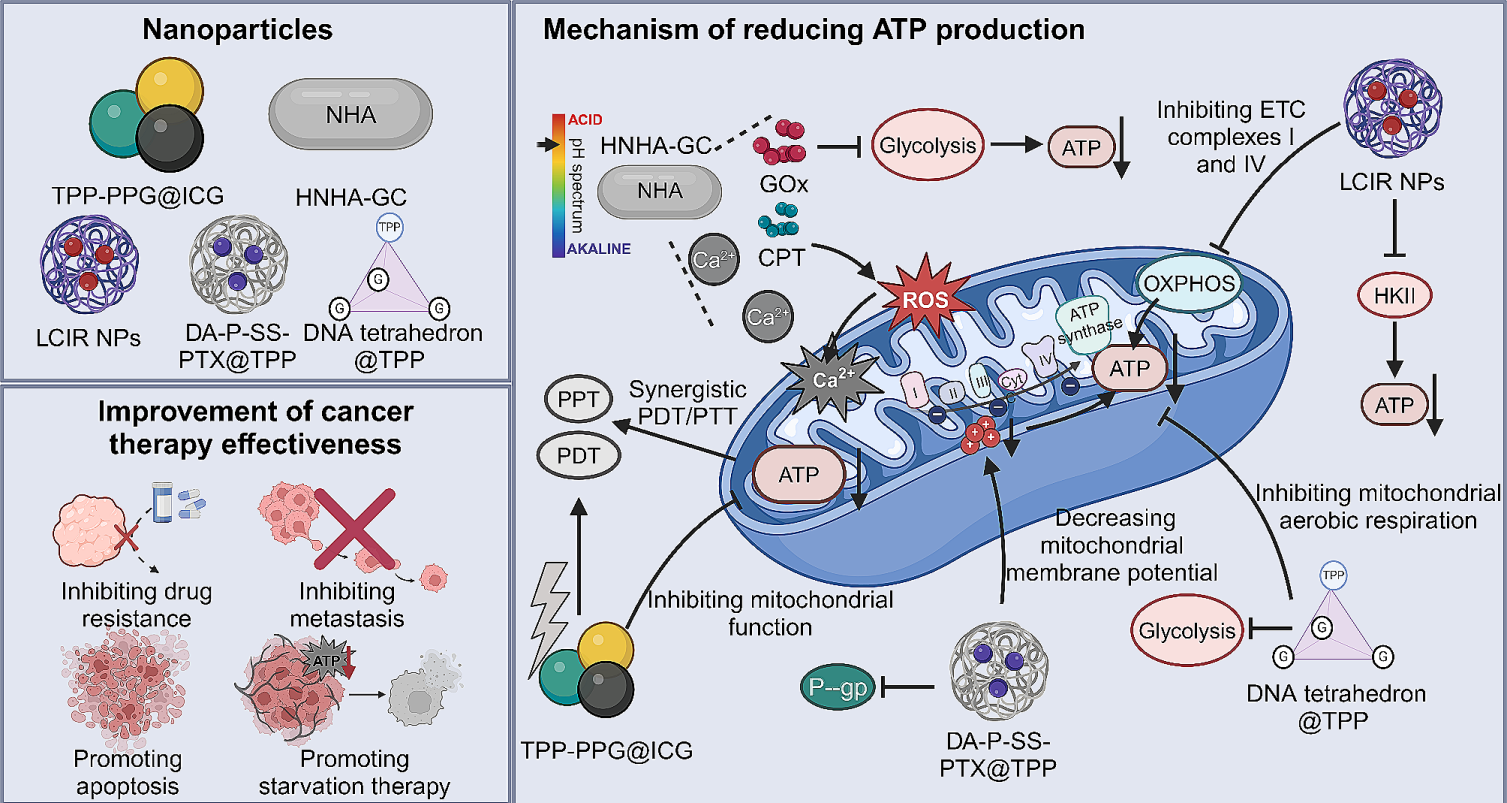
Nanomedicines enhance cancer therapy by targeting ATP production. Nanomedicines such as TPP-PPG@ICG, HNHA-GC, LCIR, DA-P-SS-PTX@TPP, and DNA tetrahedron@TPP effectively reduce ATP production by obstructing mitochondrial respiration and disrupting glycolysis, while also releasing calcium ions that induce mitochondrial dysfunction. These mechanisms collectively contribute to the inhibited growth and migration of tumor cells, thereby synergistically enhancing the efficacy of various cancer treatments

Various nanoparticles have been engineered to target ATP production in cancer cells **(**Fig. 5**)**. For instance, the TPP-PPG@ICG nanocomposite, integrating a mitochondria-targeting ligand with ICG-loaded graphene, provides synergistic photodynamic and PTT activated by near-infrared light. It disrupts ATP synthesis and mitochondrial function in cancer cells, overcoming drug resistance and leading to cell death. Proven selective and effective in experiments, TPP-PPG@ICG shows promise as a safe and potent treatment for drug-resistant osteosarcoma [142]. In addition, the HNHA-GC nanocomposite disrupts cancer cell metabolism by blocking mitochondrial respiration and glycolysis, crucial for ATP production. It releases calcium, 10-hydroxy CPT, and glucose oxidase (GOD) at tumor sites, causing mitochondrial dysfunction and inhibiting glycolysis. This trigger increased ROS and acidity, enhancing calcium overload [143]. Interestingly, the LMGC nanoparticle is designed with a liquid metal core, surface-functionalized with GOD and coated with calcium carbonate. It achieves therapeutic effects by employing GOD to disrupt glycolysis and increase oxidative stress, while calcium carbonate promotes Ca^2+^-mediated mitochondrial dysfunction. This dual approach effectively reduces ATP production and lowers heat resistance in tumor cells, thereby improving the effectiveness of PTT against tumors [144]. Notably, an abraxane-like nanoplatform named LCIR effectively depletes ATP by inhibiting mitochondrial complexes and hexokinase II, enhancing NIR-triggered photodynamic and photothermal treatments. This approach significantly reduces tumor size with minimal systemic toxicity, indicating its potential to overcome resistance in conventional cancer therapies [145]. Besides, a novel organic nanocarrier DA-P-SS-T/PTX, modified with acid-cleavable dimethylmaleic anhydride and conjugated with mitochondria-targeting TPP, demonstrates enhanced cellular uptake and specific targeting to mitochondria in tumor environments. It facilitates prolonged blood circulation, effectively targets the mitochondrial outer membrane in tumor cells, leading to decreased membrane potential and ATP levels, thereby inhibiting P-glycoprotein and curtailing both cancer drug resistance and metastasis [146]. Modified with triphenylphosphine for mitochondrial targeting, DNA tetrahedrons form aggregates in the cytoplasm, particularly targeting mitochondria. This intracellular dynamic assembly disrupts mitochondrial function, reducing aerobic respiration and glycolysis, leading to decreased ATP production. Resulting in significant inhibition of cell migration, especially in cancer cells, this approach offers a promising strategy in biomedicine for precise organelle manipulation within living cells [147].

#### Targeting TCA cycle

**Fig. 6:**
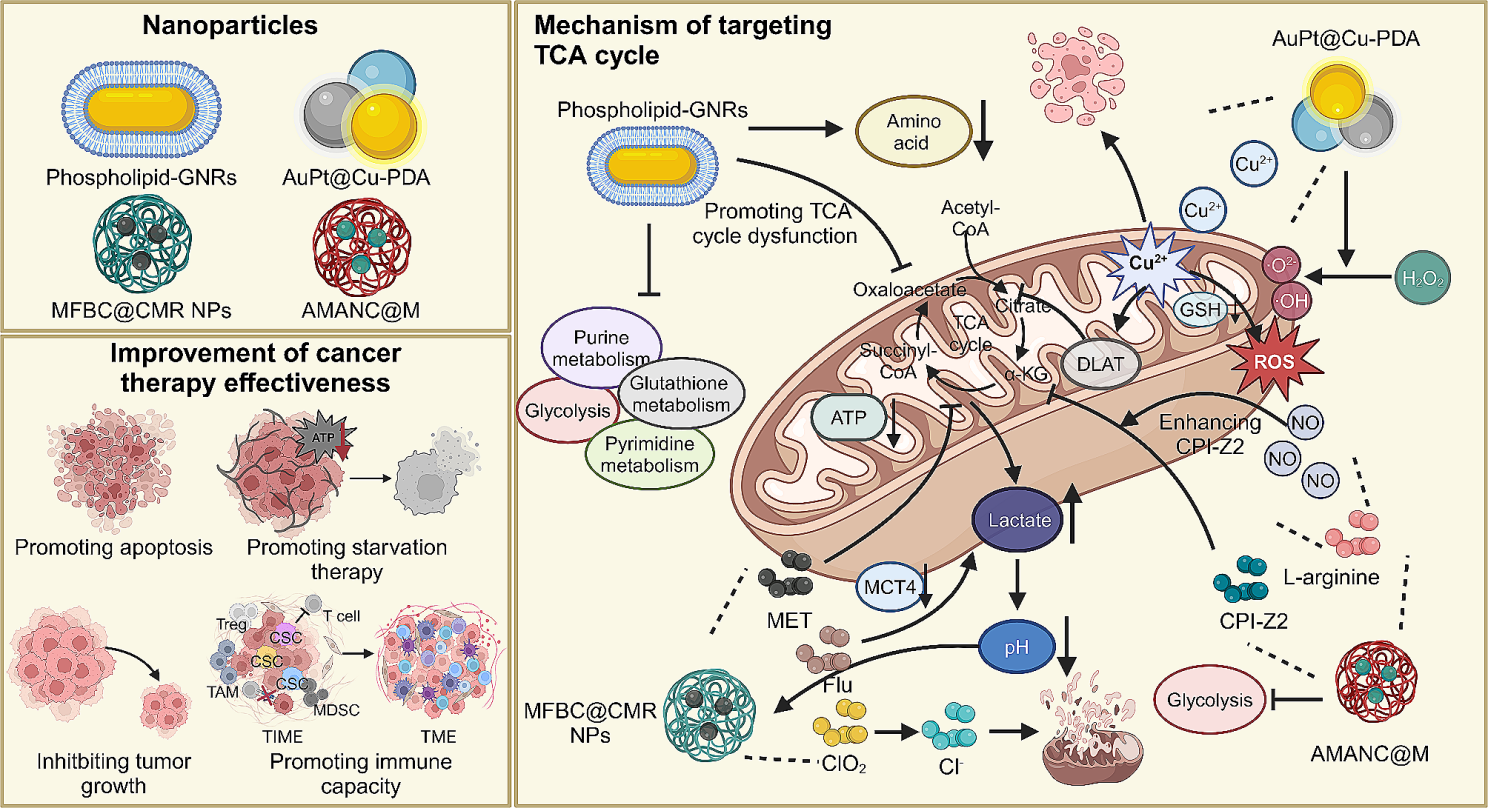
The mechanism of nanomedicines in enhancing cancer therapy through targeting the mitochondrial TCA cycle. Nanomedicines, including phospholipid-GNRs, AuPt@Cu-PDA, MFBC@CMR, and AMANC@M, disrupt the mitochondrial TCA cycle, leading to reduced cellular energy metabolism and impaired cancer cell growth. Additionally, these nanomedicines, by co-delivering other therapeutic agents, can inhibit various metabolic pathways, modulate the tumor microenvironment, and synergistically enhance the efficacy of both starvation therapy and immunotherapy

Disrupting the TCA cycle is also a promising strategy for suppressing tumor progression **(**Fig. 6**)**. For instance, phospholipid-modified gold nanorods significantly disrupt the TCA cycle in MCF-7 breast cancer cells, leading to reduced cellular energy metabolism and impaired cancer cell growth [148]. Similarly, the AuPt@Cu-PDA nanocomposite combines PTT and nanocatalytic therapy, targeting the TCA cycle in cancer cells. It disrupts copper homeostasis and induces cuproptosis, leading to the disruption of the mitochondrial TCA cycle and enhanced cancer treatment efficacy [149]. In addition, the ClO_2_-loaded nanoparticles (MFBC@CMR NPs) disrupt mitochondrial functions and TCA cycle, leading to lactate accumulation and pH reduction, triggering chlorine dioxide release. This oxidizes methionine, inhibits tumor growth, and disrupts mitochondrial Cl^−^ homeostasis, causing apoptosis. This approach combines chlorine treatment with methionine-depletion starvation therapy, offering an innovative method for tumor treatment [150]. Interestingly, AMANC@M, a redox-activatable nanoparticle, effectively targets TNBC by inhibiting glycolysis with CRISPR/Cas9 and blocking the TCA cycle with the novel drug CPI-Z2. This approach not only disrupts cancer cell metabolism but also transforms the tumor microenvironment, enhancing the efficacy of cancer therapy. Additionally, AMANC@M offers PTT and imaging capabilities, making it a promising strategy for TNBC treatment [151].

#### Other novel nanomaterials for targeting mitochondrial energy metabolism

The development of various nanotechnologies represents a significant advancement in targeting cancer cells’ mitochondrial energy metabolism, offering new avenues for effective and specific cancer therapies. For instance, developed bioreducible exosomes for targeted sonodynamic cancer therapy carry mitochondria-targeting sonosensitizers and glycolysis inhibitors. Released in tumor cells’ reducing environment, they enhance drug release and sonodynamic effects under ultrasound. This treatment disrupts mitochondria, prompts cell death, and suppresses energy metabolism, effectively inhibiting tumor growth with minimal systemic toxicity [152]. Interestingly, the CUR@DNA-FeS_2_-DA nanocomposite efficiently delivers nano-sized curcumin (CUR) to MCF-7 cancer cells’ mitochondria. Synthesized using a DNA template and poly-dopamine, it exhibits a photothermal effect and gradual CUR release in acidic environments. This nanocomposite effectively disrupts cancer cell energy metabolism by inhibiting key enzymes, offering a novel approach to target mitochondrial functions in cancer therapy [153]. Similarly, the M27-39@FA-MCNs nanoplatform combines folic acid-modified mesoporous carbon nanoparticles (FA-MCNs) with the small peptide M27-39. It offers effective targeting and bioactivity improvement for colorectal cancer (CRC) treatment. Stable in both gastric and intestinal fluids, it releases drugs efficiently, targeting mitochondrial energy metabolism and inducing apoptosis in CRC cells [154]. Furthermore, a novel nanoprodrug has been developed to simultaneously disrupt cancer cell energy metabolism and stimulate immune response. This dual-action drug, coated with F127, releases LND and NLG919 upon contact with high GSH levels. LND inhibits glycolysis by targeting hexokinase II and mitochondria, while NLG919 alleviates the immunosuppressive tumor environment [155]. Similarly, the Cy-TK-LND nanoparticles release LND in response to laser-induced oxidative stress, effectively blocking energy supply and triggering apoptosis in cancer cells. Their in vitro and in vivo efficacy, marked by a significantly lower IC50 compared to free LND and induction of tumor cell necrosis and apoptosis, demonstrates their potential as a promising, mitochondria-targeted chemotherapy strategy [156] (Table 2).

**Table 2:** Mechanisms of nanomedicines to enhance cancer therapy by disrupting mitochondrial energy metabolism

Type	Composition	Mechanism	Effects	Outcomes	Ref.
Bi-Ch	Chitosan	Inhibit OXPHOS	↓Tumor hypoxia, ↓multidrug resistance protein 1	Doxorubicin accumulation, amplify cytotoxicity	[130]
PEG-PCL liposomes	PEG-PCL, IR780 Metformin	Inhibit mitochondrial complex I	↓Respiration, overcoming hypoxia	Facilitate therapy	[131]
IR-LND@Alb NPs	IR-68, LND, Alb	Reduce PD-L1 expression	↑Self-oxygen, ↑Self-PD-L1 regulation	Facilitate therapy	[91]
PEG-GO@XN	Prenylated chalcone xanthohumol, graphene oxide	Inhibit OXPHOS	↓ATP production	Inhibit metastasis	[132]
PTX@GO-PEG-OSA	PTX, PEG, OSA, NSs	Release drugs in response to pH and temperature changes	↓ATP production	Overcome drug resistance	[133]
HM-NPs@G	Gboxin, cancer cell mitochondria hybrid membrane	Inhibit F0F1 ATPase complex V	↓OXPHOS	Inhibit tumor growth	[138]
BLG @TPGS	BBR, LND, GA	Cause mitochondrial dysfunction	↓OXPHOS, ↓glycolysis, ↓gln metabolism,	Inhibit tumor proliferation and migration	[139]
AIEPS engineered mitochondria	AIE PS Mito-AIEgen-lipid	Inhibit aerobic glycolysis	↓Anti-apoptotic protein Bcl-2	Promote cell apoptosis	[141]
TPP-PPG@ICG	TPP, PGG, ICG	Inhibit ATP synthesis and mitochondrial function	↑Synergistic photodynamic ↑PTT	Inhibit tumor progression	[142]
HNHA-GC nanocomposite	Gox, HA, CPT, NHA nanorods	Block mitochondrial respiration and glycolysis	↑ROS, ↑acidity	Disrupt cancer cell metabolism	[143]
LMGC NPs	LMGC, GOx, calcium carbonate	Disrupt glycolysis, mitochondrial dysfunction	↓ATP production, ↓ heat resistance	Improve the effectiveness of PTT	[144]
LCIR nanoplatform	CDM, LND-TPP, IR780 albumin nanoparticles	Inhibit mitochondrial complexes and hexokinase II	↑Photodynamic treatments, ↑photothermal treatments	Reduce tumor size	[145]
DA-P-SS-T/PTX	PTX, DA, PTT	Target mitochondrial outer membrane, inhibit P-glycoprotein	↓Membrane potential, ↓ATP	Curtail drug resistance and metastasis	[146]
DNA tetrahedrons	DNA	Mitochondrial dysfunction, reduce aerobic respiration and glycolysis	↓ATP production	Inhibition of cell migration	[147]
Phospholipid-GNRs	Phospholipid, gold nanorods	Decrease energy metabolisms metabolic intermediates and end-products	↓TCA cycle, ↓glycolytic activity, redox state imbalance	Inhibit cancer cell growth and proliferation	[148]
AuPt@Cu-PDA	AuPt, Cu, PDA	Disrupt copper homeostasis, produce ROS, deplete GSH, produce O_2_	↓TCA, ↑ROS, ↑oxidative stress, reverse hypoxia, ↑cuproptosis, reverse TIME	Kill cancer cells, enhance efficacy of PTT	[149]
MFBC@CMR NPs	Fluvastatin sodium, metformin, bupivacaine, ClO_2_, CaSiO_3_, MnO_2_-arginine-glycine-aspatic acid	Mitochondrial dysfunction, produce lactate and Cl^−^	↓TCA, ↓membrane potential, mitochondrial damage, ↑apoptosis	Kill cancer cells, enhance starvation therapy efficacy	[150]
AMANC@M	Au@MSN NPs, CRISPR/Cas9 system, CPI-Z2, L-Arg	Block TCA cycle, inhibit LDH	↓TCA, ↓glycolysis, reverse TIME	Enhance PPT therapy	[151]
Bioreducible exosomes	Exosomes	Carry mitochondria-targeting sonosensitizers and glycolysis inhibitors	Enhance drug release and sonodynamic effects	Inhibit tumor growth	[152]
CUR@DNA-FeS_2_-DA	DNA-FeS_2_-DA CUR	Release CUR, Exhibit a photothermal effect	↓Energy metabolism	Facilitate therapy	[153]
M27-39@FA-MCNs	FA-MCNs M27-39	Disrupt mitochondrial energy metabolism	↑Cell apoptosis	Inhibit tumor growth	[154]
GSH-responsive dimeric prodrug	LND NLG919, F127	Decrease hexokinase II, destroy mitochondria, reduce kynurenine	↓Energy metabolism, ↑immune response	Inhibit tumor growth	[155]
Cy-TK-LND NPs	Cy, TK, LND	Facilitate the release of free LND into mitochondria	↓Mitochondria damage, ↑apoptosis pathway	Promote cell apoptosis	[156]

### Targeting FAO

**Fig. 7:**
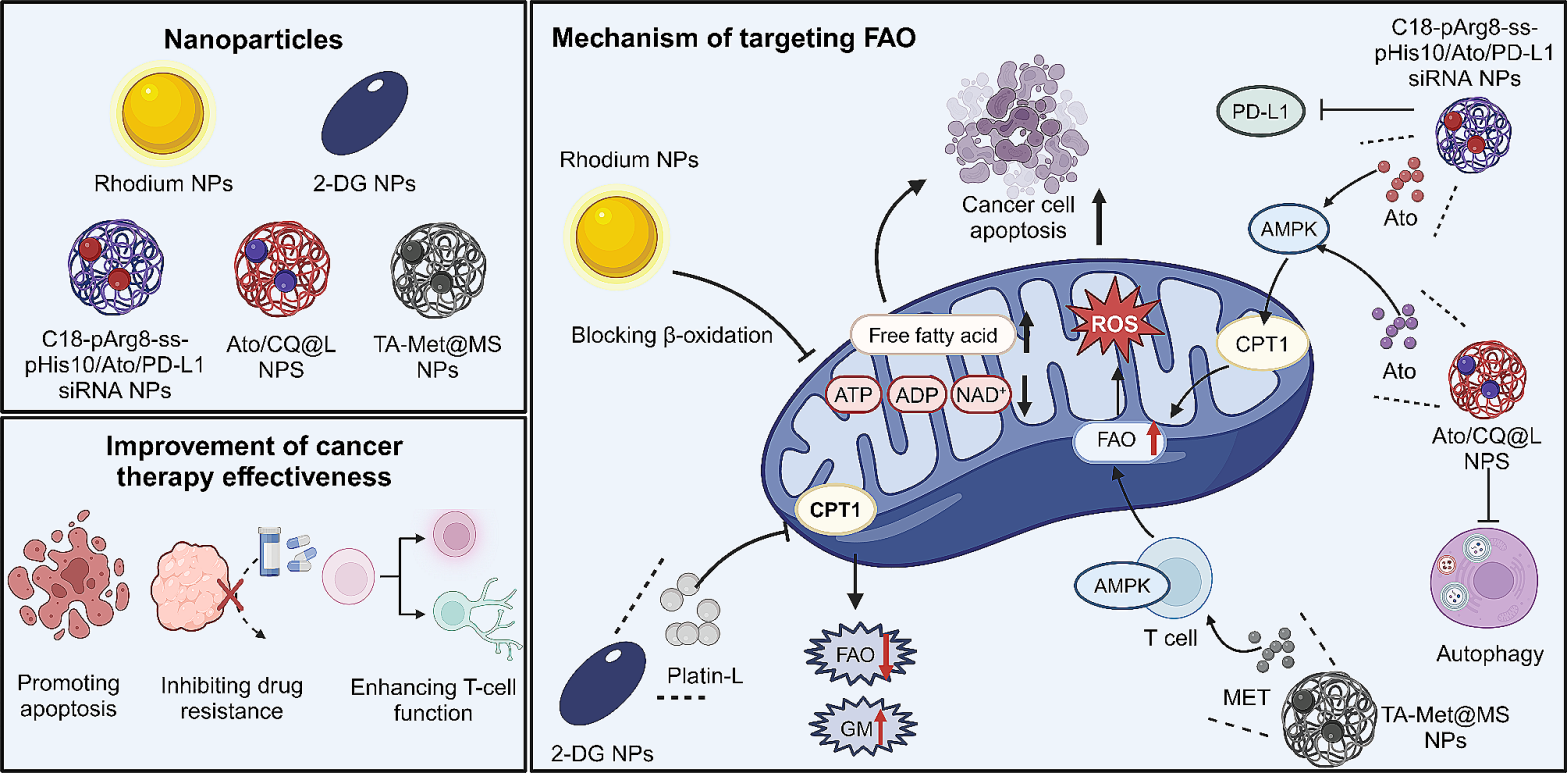
Mechanisms of nanomedicines to enhance cancer therapy by targeting mitochondrial FAO Nanomedicines targeting mitochondrial FAO, including rhodium NPs, 2-DG, C18-pArg8-ss-pHis10/Ato/PD-L1 siRNA, Ato/cq@L, and TA-Met@MS, demonstrate diverse therapeutic actions. Rhodium NPs and 2-DG reduce the energy supply in tumor cells by inhibiting FAO, resulting in cell apoptosis due to the accumulation of free fatty acids. Moreover, suppression of FAO heightens tumor cells’ reliance on glucose metabolism, enhancing the efficacy of cisplatin in drug-resistant tumors. Alternatively, activation of the AMPK pathway to promote FAO not only aids in ROS production, leading to tumor cell apoptosis, but also improves the differentiation and survival of CD8^+^ T cells, augmenting immune function

FAO plays a crucial role in cancer cells, serving as a vital energy source for their growth and proliferation. Targeting FAO with nanomaterials presents a promising strategy for cancer treatment, as it disrupts the energy supply essential for tumor cell survival and progression **(**Fig. 7**)**. For instance, rhodium nanoparticles in PDT target β-oxidation in cancer cells, disrupting their energy metabolism. This leads to decreased levels of ATP, ADP, and NAD^+^, and an accumulation of free fatty acids, effectively inducing apoptosis by altering apoptotic factors [157]. Similarly, a cascade-responsive 2-DG nanocapsule delivery system has been developed to treat GBM by targeting both glycolysis and FAO. It combines an anti-vascular endothelial growth factor receptor 2 monoclonal antibody with CPT1C siRNA, effectively penetrating the BBB and reducing GBM cell energy supply and angiogenesis. Platin-L, a cisplatin-based prodrug, targets FAO in prostate cancer cells by inhibiting CPT1A, shifting them to a glucose-dependent state. Incorporated into a targeted oral nanoformulation, it demonstrates potential for treating cisplatin-resistant cancer forms by exploiting its unique FAO inhibitory property [158]. While inhibiting FAO can cut off the energy supply of tumor cells, some studies have also shown that appropriately activating the FAO pathway to produce ROS can also achieve the goal of inhibiting tumors. For example, a nanoplex co-encapsulating atorvastatin (Ato) and PD-L1 siRNA targets FAO in cancer cells, activating AMPK and boosting mitochondrial FAO. This leads to a self-amplifying cycle of ROS production, resulting in effective tumor cell death and improved anti-tumor efficacy [159]. Likewise, liposome nanoparticles (Ato/CQ@L), combining Ato and chloroquine (CQ), enhance FAO in cancer cells to treat drug-resistant tumors. Ato boosts ROS production through FAO, while CQ inhibits autophagy, leading to increased apoptosis in tumor cells, effectively addressing the challenges of conventional ROS-based therapies [160]. Notably, a tumor vaccine vector (TA-Met@MS) using PLGA microspheres to deliver tumor antigen, Met, and hollow gold nanospheres shifts cellular metabolism from glycolysis to FAO via Met-induced AMPK activation. This enhances the differentiation and survival of memory CD8^+^ T cells, offering a novel approach for cancer therapy [161].

### Targeting gln metabolism

Cancer cells often exhibit a marked dependence on exogenous Gln, leveraging it predominantly as an anaplerotic substrate for the TCA cycle, rather than as a building block for protein synthesis. This Gln addiction is underscored by increased activity of the enzyme GLS [162]. In a notable study, Andrew et al. uncovered that tumor cells with compromised mitochondrial oxidative capacities utilized a Gln-dependent reductive carboxylation pathway. Facilitated by both mitochondrial and membrane-bound isoforms of NADP^+^/NADPH-dependent isocitrate dehydrogenase, this alternative metabolic route generates citrate and its downstream products, notably acetyl-CoA for lipid biosynthesis. This pathway also replenishes four-carbon intermediates essential for the synthesis of other TCA cycle metabolites and biomass precursors. Predominantly active in rapidly proliferating malignant cells with mutations in the ETC complex I or III, renal carcinoma cells derived from fumarate hydratase mutations, and cells with inhibited mitochondrial ETC function, this Gln-dependent reductive pathway signifies a major metabolic adaptation in tumor cell physiology [163, 164].

**Fig. 8:**
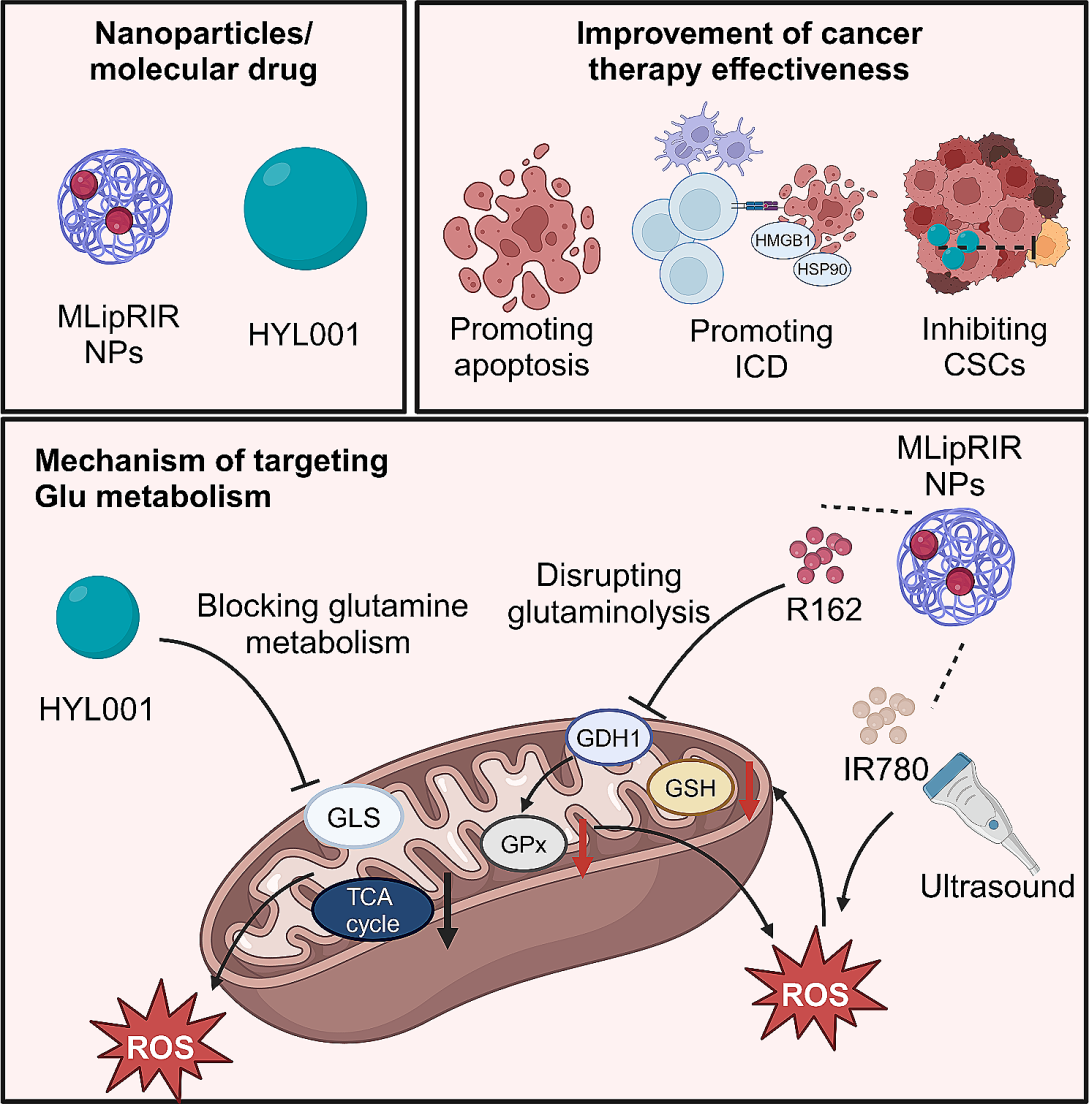
Mechanisms of nanomedicine and HYL001 in enhancing cancer therapy by inhibiting mitochondrial glutamine metabolism. MlipRIR, through the release of R162, disrupts this metabolism via GDH1, leading to increased ROS and redox imbalance, which induces tumor cell apoptosis and immunogenic cell death (ICD), thereby augmenting anti-tumor immunity. Simultaneously, IR780 incorporated in MlipRIR generates high ROS levels under ultrasound stimulation, resulting in GSH depletion and GPx inactivation, and ultimately causing severe tumor cell ferroptosis. HYL001, by targeting GLS, similarly impedes glutamine metabolism

Current research on using nanomaterials to target Gln metabolism in cancer treatment is limited **(**Fig. 8**)**. MLipRIR nanoparticles, which are liposome-encapsulated and incorporate R162 and IR780, specifically target mitochondrial dysfunction to induce ferroptosis and immunogenic cell death in cancer cells. Activated by ultrasound, they impair glutaminolysis and increase ROS levels, disrupting redox homeostasis [165]. Interestingly, HYL001, a novel LND derivative, effectively targets cancer stem cells by inhibiting GLS, thereby disrupting Gln metabolism and amplifying mitochondrial stress. Therefore, encapsulating HYL001 within nanomaterials could enhance its therapeutic potential [166].

### Targeting calcium overload to mediate mitochondrial metabolism

**Fig. 9:**
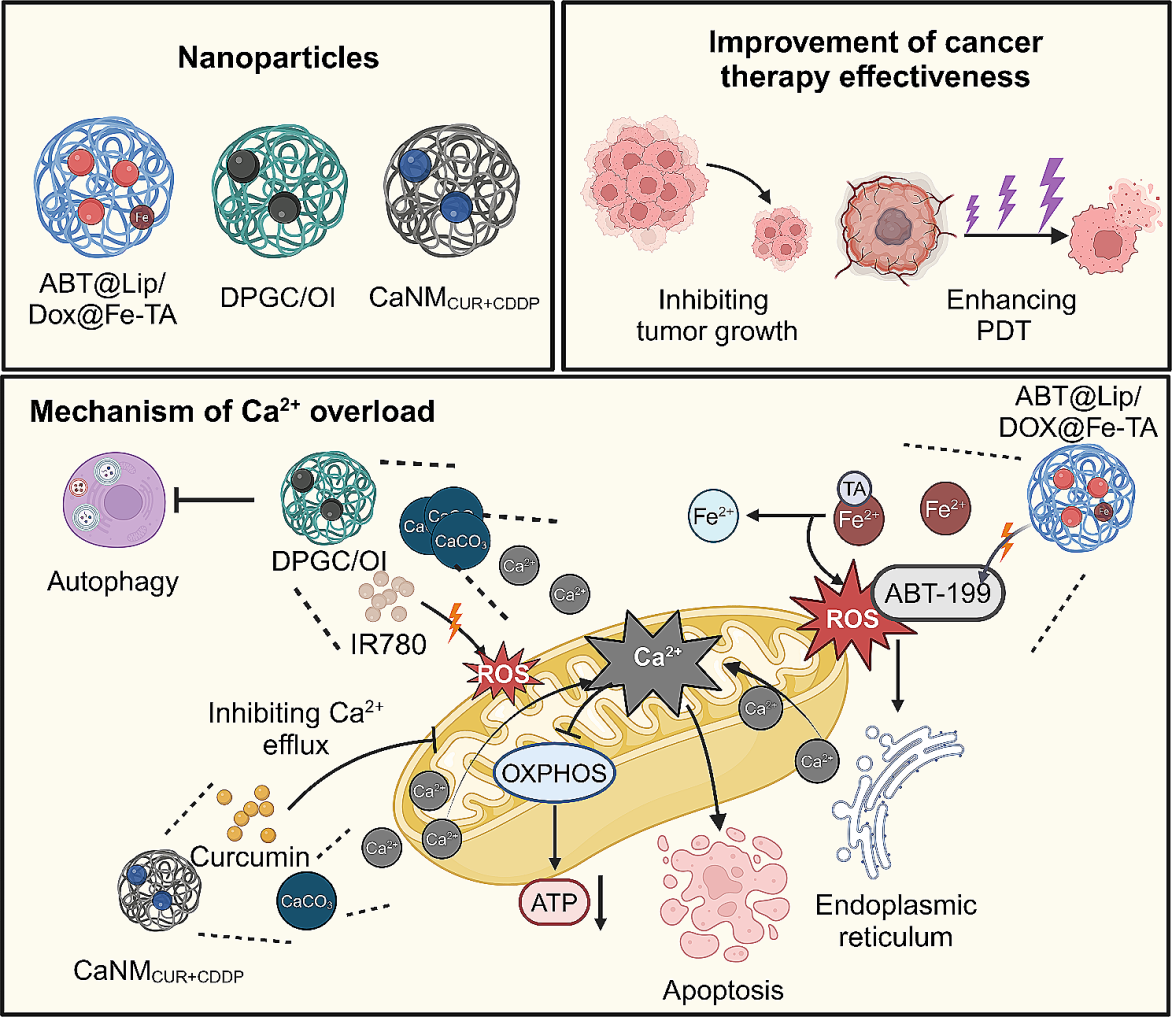
Enhancing cancer treatment efficacy through mitochondrial calcium overload mediated by nanomedicines. Nanomedicines including ABT@Lip/Dox@Fe-TA, DPGC/OI, and CaNM_CUR + CDDP_ induce mitochondrial calcium overload by facilitating the release of exogenous calcium ions, enhancing the transport of endogenous calcium ions from the endoplasmic reticulum to mitochondria, and inhibiting mitochondrial calcium ion efflux. This process leads to mitochondrial dysfunction, elevated ROS production, and the initiation of cell apoptosis, thereby effectively eradicating tumor cells

In cancer therapy, inducing calcium overload in cancer cells can be an effective strategy. This approach aims to disrupt mitochondrial metabolism in cancer cells, as calcium overload interferes with the normal functions of mitochondria, impairing the ETC and OXPHOS, thereby reducing ATP production. Additionally, excessive calcium can trigger mitochondrial-mediated apoptosis pathways, increasing cancer cell death. Therefore, targeted elevation of intracellular calcium levels through nanomaterials or drug delivery systems may enhance the efficacy of cancer treatments **(**Fig. 9**)** [167, 168]. For instance, a calcium ion nanomodulator combining cisplatin and CUR within calcium carbonate nanoparticles enhances cancer therapy. Targeting mitochondria, it releases calcium ions, disrupting mitochondrial function and boosting tumor inhibition. This nanomodulator also allows for tumor tracking through fluorescence and photoacoustic imaging, offering a novel strategy for bioimaging-guided, mitochondria-focused cancer treatment [169]. Additionally, a novel nanoplatform named ABT-199@liposomes/Dox@FeIII-tannic acid is designed to transport endogenous calcium ions from the endoplasmic reticulum to mitochondria, increasing intramitochondrial calcium levels. This occurs through a cascade release mechanism in the tumor’s acidic environment, enhancing mitochondrial dysfunction and tumor inhibition [170]. Importantly, the biodegradable nanocomposite material DPGC/OI, encapsulating IR780 and obatoclax, represents a novel strategy to enhance PDT. It induces mitochondrial Ca^2+^ overload through calcium phosphate, leading to mitochondrial dysfunction and increased ROS production. Concurrently, obatoclax inhibits cancer cell autophagy, countering resistance to PDT and boosting its effectiveness with minimal toxicity [171] **(**Table 3**)**.

**Table 3:** Mechanisms of nanomedicines to enhance cancer therapy by targeting mitochondrial FAO, glutamine metabolism and Ca^2+^ overload

Type	Composition	Mechanisms	Effects	Outcomes	Ref.
RhNPs	PVP, RhCl_3_	Block β-oxidation	↓ATP, ↓ADP, ↓NAD^+^, ↑free fatty acids	Kill cancer cells	[157]
2-DG/aV-siCPT1C NC	2-DG, aV, siCPT1C	Suppress FAO	↓Glycolysis, ↓FAO, ↓angiogenesis	Inhibit tumor growth	[158]
LNPs	C18-pArg8-ss-pHis10, PD-L1 siRNA, Ato	Activate AMPK, suppress FAO	↑ROS, ↑FAO, ↓triglyceride synthesis	Kill cancer cells, inhibit tumor growth, reprogram TME	[159]
Ato/CQ@L	Ato, CQ	Inhibit autophagy, upregulate CPT1	↑ROS, ↑CPT1, ↑FAO	Kill cancer cells	[160]
TAMet@MS	TA, Met, HAuNS, PLGA	Activate FAO, activate AMPK	↑Effector T cell, ↑FAO	Enhance the differentiation and survival of memory CD8 + T cell	[161]
MLipRIR NPs	R162, IR780	Disrupt glutaminolysis pathway, downregulate GPx activity	↑ROS, ↓GSH	Kill cancer cells, activate antitumor immunity	[165]
HYL001	LND	Inhibit GLS, disrupt Gln metabolism	↑Mitochondrial stress	Tumor inhibition	[166]
CaNM_CUR+CDDP_	CDDP, CUR, CaCO_3_	Inhibit Ca^2+^efflux, destruct mitochondria	↑Ca^2+^	Tumor inhibition	[169]
ABT@Lip/Dox@Fe-TA	Lip, DPPC, DSPC, DSPE-PEG_2k_-NH_2,_ Dox, NaOH	Mitochondrial Ca^2+^overload	↑ROS, ↑Ca^2+^	Mitochondrial dysfunction, tumor inhibition	[170]
DPGC/OI	CP, DSPE, PEG, DPG, IR780, Obatoclax	Mitochondrial Ca^2+^overload	↑ROS, ↑Ca^2+^	Enhance PDT, inhibit cancer cell autophagy	[171]

## Future challenges and prospects

As scientific research advances, nanomedicine is increasingly unlocking possibilities for precise tumor treatments, with a growing number of nanomedicines under development and testing [172, 173]. Furthermore, the utilization of nanotechnology for mitochondrial targeting offers a more precise means of modulating cellular energy metabolism, thereby effectively intervening in disease progression [174]. Additionally, the design of nanomedicines enables them to efficiently traverse biological barriers, such as the BBB. This capability opens up therapeutic possibilities for treating lesions that are typically inaccessible to conventional drugs [175, 176]. Moreover, the highly customizable nature of nanomedicines allows for the tailored adjustment of drug release rates and dosages according to individual patient requirements. This personalization can potentially minimize side effects and enhance therapeutic outcomes [177]. However, the deployment of nanomedicines in targeting mitochondrial metabolism for cancer treatment presents several challenges.

Firstly, achieving specific targeting of cancer cell mitochondria while sparing normal cells presents a significant challenge due to the ubiquitous presence of mitochondria in all cell types. This necessitates the development of nanomedicines with precise targeting strategies, involving the creation of specific ligands that exploit unique mitochondrial surface proteins or metabolic signatures exclusive to tumor cells [178, 179]. Future research is set to focus on designing nanomedicines responsive to distinct tumor microenvironment characteristics, such as altered pH levels, reductive states, or enzymatic activities prevalent in cancerous tissues. By utilizing differential expression of mitochondrial markers or the distinctive metabolic pathways in cancer cells compared to normal cells, these nanomedicines can be tailored for controlled release and targeted action [180–182]. Advances in this field require a comprehensive understanding of both tumor biology and the physicochemical properties of nanocarriers, aiming to create stimuli-responsive systems that maximize therapeutic efficacy and minimize off-target effects. This approach promises to revolutionize the treatment of multidrug-resistant cancers, ushering in a new era of precision oncology.

Secondly, although mitochondrial metabolism plays a crucial role in tumorigenesis and has potential as a therapeutic strategy for cancer, translating this into clinical practice remains challenging. Several compounds inhibiting mitochondrial metabolic enzymes, including etomoxir and BPTES, have demonstrated promising in vitro results [183, 184]. However, only a few of these compounds are currently in the early stages of clinical trials. The limited progress in clinical translation can be attributed to cancer cells undergoing metabolic reprogramming or metabolic coupling, allowing them to overcome nutrient-deprived environments and promote proliferation [7, 185]. Additionally, this adaptive capacity of cancer cells is further complicated by their inherent genetic and epigenetic variability [186, 187]. To counteract this, current research is focusing intensively on leveraging nanomedicines to interrupt and reprogram cancer cell metabolism and signaling pathways. The objective is to specifically target and dismantle the cellular mechanisms that confer drug resistance [8]. Furthermore, an emerging strategy involves the integration of nanomedicines with a spectrum of conventional and novel therapeutic modalities. This includes the combination of nanomedicines with established treatments such as chemotherapy and targeted therapy, as well as immunotherapy. The rationale behind this multifaceted strategy is to not only enhance the overall therapeutic efficacy but also to pre-emptively tackle the mechanisms that lead to drug resistance. By orchestrating a coordinated attack on cancer cells from multiple fronts, this approach seeks to exploit the full potential of nanomedicines while simultaneously addressing the complex and dynamic nature of cancer cell resistance [82].

Thirdly, mitochondria consist of substructures including OMM, IMM, IMS, and MM, each with distinct functions, particularly in their membrane proteins. Designing nanocarriers that specifically target key metabolic points in mitochondria requires an in-depth understanding of the biological functions of these substructures. Moreover, the inherent complexity of mitochondria presents challenges in precisely elucidating the mechanisms of certain diseases involving mitochondrial processes, which hinders the application of nanomedicine for targeted therapy [188]. A prime example is the role of the mitochondrial permeability transition pore complex (MPT) in the pathogenesis of cardiovascular diseases. MPT results from the activity of a supramolecular entity called the permeability transition pore complex (PTPC), which aggregates at the interface between the mitochondrial inner and outer membranes. However, the exact molecular composition of PTPC remains undetermined, and several other aspects of PTPC biology, including its potential connection with the F0F1 ATP synthase, are still subjects of intense debate [188, 189]. This complexity underscores the need for continued research to better understand mitochondrial biology for the development of more effective mitochondria-targeted nanotherapeutics.

Fourthly, mitochondria engage in complex functional and material interactions with other cellular organelles such as the endoplasmic reticulum and the cytoskeleton [190–192]. There is a possibility that these organelles might exhibit compensatory functions, potentially influencing the effectiveness of nanomedicines targeting mitochondrial metabolism. Solely targeting mitochondria might not suffice to achieve the desired metabolic regulatory effects. Therefore, a significant challenge lies in achieving coordinated targeting of multiple cellular components. This necessitates a comprehensive understanding of the intricate cellular network and the interplay between various organelles. Developing nanomedicines that can simultaneously target multiple cellular sites requires not only precision in design but also a deeper insight into the cellular mechanisms governing disease pathology. Addressing this issue is critical for enhancing the efficacy of nanomedicine-based therapies, ensuring that they can effectively modulate complex metabolic pathways within the cellular environment.

Lastly, the development of nanomaterials with enhanced safety profiles is crucial in addressing the challenges of unknown long-term toxicity and immune responses associated with nanomedicines. Given the unique interactions of nanoscale materials within biological systems, comprehensive evaluations of biocompatibility, toxicity, and immunogenicity are essential. It is important to design biodegradable and non-immunogenic nanomaterials that naturally decompose after their therapeutic action, thereby reducing the risks of prolonged toxicity. Such materials are tailored to ensure their degradation products are harmless and can be effectively metabolized or excreted. Moreover, understanding the immune system’s interaction with these nanomaterials is critical, as it significantly impacts both the effectiveness and potential adverse effects of the therapy. This direction in nanomaterial development aims to harness the benefits of nanomedicine in clinical applications while mitigating safety concerns, thereby striking a balance between therapeutic efficiency and patient safety [193, 194].

## Conclusions

In summary, mitochondria are central to cellular energy processes, and their dysfunction is closely associated with cancer development and treatment outcomes. The focus on using multifunctional nanosystems to target mitochondrial metabolism in tumor cells represents a promising approach in cancer therapy. This innovative direction exploits the specific metabolic vulnerabilities of cancer cells, potentially leading to more effective and targeted treatments. Advances in nanomedicine targeting mitochondrial pathways may revolutionize cancer therapy and have implications for managing other diseases characterized by mitochondrial dysregulation.

## Data Availability

No datasets were generated or analysed during the current study.

## Abbreviations

OMM: Outer mitochondrial membrane
IMS: Intermembrane space
IMM: Inner mitochondrial membrane
MM: Mitochondrial matrix
Acetyl-CoA: Acetyl coenzyme A
TCA: Tricarboxylic acid
OXPHOS: Oxidative phosphorylation
FAO: Fatty acid oxidation
Gln: Glutamine
ROS: Reactive oxygen species
MtDNA: Mitochondrial DNA
ETC: Electron transport chain
CPT: Carnitine palmitoyltransferase
Glu: Glutamate
GSH: Glutathione
GLS: Glutaminase
GDH: Glutamate dehydrogenase
RNS: Reactive nitrogen species
PTT: Photothermal therapy
PDT: Photodynamic therapy

## References

[1] Antonicka H, Lin ZY, Janer A, Aaltonen MJ, Weraarpachai W, Gingras AC (2020). A high-density human mitochondrial proximity Interaction Network. Cell Metab.

[2] Nunnari J, Suomalainen A (2012). Mitochondria: in sickness and in health. Cell.

[3] Bozi LHM, Campos JC, Zambelli VO, Ferreira ND, Ferreira JCB (2020). Mitochondrially-targeted treatment strategies. Mol Aspects Med.

[4] Wallace DC (1999). Mitochondrial diseases in man and mouse. Science.

[5] Amorim JA, Coppotelli G, Rolo AP, Palmeira CM, Ross JM, Sinclair DA (2022). Mitochondrial and metabolic dysfunction in ageing and age-related diseases. Nat Rev Endocrinol.

[6] Zhang X, Xie F, Ma S, Ma C, Jiang X, Yi Y (2023). Mitochondria: one of the vital hubs for molecular hydrogen’s biological functions. Front Cell Dev Biol.

[7] Martinez-Outschoorn UE, Peiris-Pages M, Pestell RG, Sotgia F, Lisanti MP (2017). Cancer metabolism: a therapeutic perspective. Nat Rev Clin Oncol.

[8] Jin P, Jiang J, Zhou L, Huang Z, Nice EC, Huang C (2022). Mitochondrial adaptation in cancer drug resistance: prevalence, mechanisms, and management. J Hematol Oncol.

[9] Scagliola A, Mainini F, Cardaci S (2020). The Tricarboxylic Acid cycle at the crossroad between Cancer and Immunity. Antioxid Redox Signal.

[10] Murphy MP, O’Neill LAJ (2018). Krebs cycle reimagined: the emerging roles of Succinate and Itaconate as Signal transducers. Cell.

[11] Martinez-Reyes I, Chandel NS (2020). Mitochondrial TCA cycle metabolites control physiology and disease. Nat Commun.

[12] Pokharel MD, Marciano DP, Fu P, Franco MC, Unwalla H, Tieu K (2023). Metabolic reprogramming, oxidative stress, and pulmonary hypertension. Redox Biol.

[13] Ryan DG, Murphy MP, Frezza C, Prag HA, Chouchani ET, O’Neill LA (2019). Coupling Krebs cycle metabolites to signalling in immunity and cancer. Nat Metab.

[14] Murphy MP, Chouchani ET (2022). Why succinate? Physiological regulation by a mitochondrial coenzyme Q sentinel. Nat Chem Biol.

[15] Sazanov LA (2015). A giant molecular proton pump: structure and mechanism of respiratory complex I. Nat Rev Mol Cell Biol.

[16] Vercellino I, Sazanov LA (2022). The assembly, regulation and function of the mitochondrial respiratory chain. Nat Rev Mol Cell Biol.

[17] Neupane P, Bhuju S, Thapa N, Bhattarai HK (2019). ATP synthase: structure, function and inhibition. Biomol Concepts.

[18] Gupta N, Verma K, Nalla S, Kulshreshtha A, Lall R, Prasad S. Free radicals as a double-edged Sword: the Cancer Preventive and therapeutic roles of Curcumin. Molecules. 2020;25(22). 10.3390/molecules25225390 PMC7698794 33217990

[19] Moldogazieva NT, Lutsenko SV, Terentiev AA (2018). Reactive oxygen and Nitrogen species-Induced protein modifications: implication in carcinogenesis and anticancer therapy. Cancer Res.

[20] Barrera G (2012). Oxidative stress and lipid peroxidation products in cancer progression and therapy. ISRN Oncol.

[21] Hajam YA, Rani R, Ganie SY, Sheikh TA, Javaid D, Qadri SS et al. Oxidative stress in Human Pathology and Aging: Molecular mechanisms and perspectives. Cells. 2022;11(3). 10.3390/cells11030552 PMC8833991 35159361

[22] Weindel CG, Martinez EL, Zhao X, Mabry CJ, Bell SL, Vail KJ et al. Mitochondrial ROS promotes susceptibility to infection via gasdermin D-mediated necroptosis. Cell. 2022;185(17):3214-31 e23. 10.1016/j.cell.2022.06.038 PMC9531054 35907404

[23] Park MW, Cha HW, Kim J, Kim JH, Yang H, Yoon S (2021). NOX4 promotes ferroptosis of astrocytes by oxidative stress-induced lipid peroxidation via the impairment of mitochondrial metabolism in Alzheimer’s diseases. Redox Biol.

[24] Yang ZJ, Zhao CL, Liang WQ, Chen ZR, Du ZD, Gong SS (2024). ROS-induced oxidative stress and mitochondrial dysfunction: a possible mechanism responsible for noise-induced ribbon synaptic damage. Am J Transl Res.

[25] Zorova LD, Popkov VA, Plotnikov EY, Silachev DN, Pevzner IB, Jankauskas SS (2018). Mitochondrial membrane potential. Anal Biochem.

[26] Houten SM, Violante S, Ventura FV, Wanders RJ (2016). The Biochemistry and Physiology of mitochondrial fatty acid beta-oxidation and its genetic disorders. Annu Rev Physiol.

[27] Grevengoed TJ, Klett EL, Coleman RA (2014). Acyl-CoA metabolism and partitioning. Annu Rev Nutr.

[28] Kim JA. Peroxisome metabolism in Cancer. Cells. 2020;9(7). 10.3390/cells9071692 PMC7408135 32674458

[29] Martinez-Outschoorn UE, Peiris-Pages M, Pestell RG, Sotgia F, Lisanti MP (2017). Cancer metabolism: a therapeutic perspective. Nat Rev Clin Oncol.

[30] Panieri E, Telkoparan-Akillilar P, Suzen S, Saso L. The NRF2/KEAP1 Axis in the regulation of Tumor Metabolism: mechanisms and therapeutic perspectives. Biomolecules. 2020;10(5). 10.3390/biom10050791 PMC7277620 32443774

[31] Garcia-Bermudez J, Williams RT, Guarecuco R, Birsoy K (2020). Targeting extracellular nutrient dependencies of cancer cells. Mol Metab.

[32] Mates JM, Campos-Sandoval JA, Santos-Jimenez JL, Marquez J (2019). Dysregulation of glutaminase and glutamine synthetase in cancer. Cancer Lett.

[33] Zhao RZ, Jiang S, Zhang L, Yu ZB (2019). Mitochondrial electron transport chain, ROS generation and uncoupling (review). Int J Mol Med.

[34] Zorov DB, Juhaszova M, Sollott SJ (2014). Mitochondrial reactive oxygen species (ROS) and ROS-induced ROS release. Physiol Rev.

[35] Sies H, Jones DP (2020). Reactive oxygen species (ROS) as pleiotropic physiological signalling agents. Nat Rev Mol Cell Biol.

[36] Lennicke C, Rahn J, Lichtenfels R, Wessjohann LA, Seliger B (2015). Hydrogen peroxide - production, fate and role in redox signaling of tumor cells. Cell Commun Signal.

[37] Raimondi V, Ciccarese F, Ciminale V (2020). Oncogenic pathways and the electron transport chain: a dangeROS liaison. Br J Cancer.

[38] Shadel GS, Horvath TL (2015). Mitochondrial ROS signaling in organismal homeostasis. Cell.

[39] Parekh A, Das S, Parida S, Das CK, Dutta D, Mallick SK (2018). Multi-nucleated cells use ROS to induce breast cancer chemo-resistance in vitro and in vivo. Oncogene.

[40] Tyagi A, Haq S, Ramakrishna S (2021). Redox regulation of DUBs and its therapeutic implications in cancer. Redox Biol.

[41] Zhang J, Wang X, Vikash V, Ye Q, Wu D, Liu Y (2016). ROS and ROS-Mediated Cellular Signaling. Oxid Med Cell Longev.

[42] Sainero-Alcolado L, Liano-Pons J, Ruiz-Perez MV, Arsenian-Henriksson M (2022). Targeting mitochondrial metabolism for precision medicine in cancer. Cell Death Differ.

[43] Pavlova NN, Thompson CB (2016). The emerging Hallmarks of Cancer Metabolism. Cell Metab.

[44] Kodama M, Oshikawa K, Shimizu H, Yoshioka S, Takahashi M, Izumi Y (2020). A shift in glutamine nitrogen metabolism contributes to the malignant progression of cancer. Nat Commun.

[45] Kawamura T, Takehora Y, Hori N, Takakura Y, Yamaguchi N, Takano H (2022). VGLL3 increases the dependency of cancer cells on de novo nucleotide synthesis through GART expression. J Cell Biochem.

[46] Bian X, Liu R, Meng Y, Xing D, Xu D, Lu Z. Lipid metabolism and cancer. J Exp Med. 2021;218(1). 10.1084/jem.20201606 PMC7754673 33601415

[47] Padanad MS, Konstantinidou G, Venkateswaran N, Melegari M, Rindhe S, Mitsche M (2016). Fatty acid oxidation mediated by Acyl-CoA synthetase long chain 3 is required for mutant KRAS lung tumorigenesis. Cell Rep.

[48] Wright HJ, Hou J, Xu B, Cortez M, Potma EO, Tromberg BJ (2017). CDCP1 drives triple-negative breast cancer metastasis through reduction of lipid-droplet abundance and stimulation of fatty acid oxidation. Proc Natl Acad Sci U S A.

[49] Bi J, Mischel PS (2019). Acyl-CoA-Binding protein fuels gliomagenesis. Cell Metab.

[50] Ma Y, Temkin SM, Hawkridge AM, Guo C, Wang W, Wang XY (2018). Fatty acid oxidation: an emerging facet of metabolic transformation in cancer. Cancer Lett.

[51] Shi J, Fu H, Jia Z, He K, Fu L, Wang W (2016). High expression of CPT1A predicts adverse outcomes: a potential therapeutic target for Acute myeloid leukemia. EBioMedicine.

[52] Liu PP, Liu J, Jiang WQ, Carew JS, Ogasawara MA, Pelicano H (2016). Elimination of chronic lymphocytic leukemia cells in stromal microenvironment by targeting CPT with an antiangina drug perhexiline. Oncogene.

[53] Tang M, Dong X, Xiao L, Tan Z, Luo X, Yang L (2022). CPT1A-mediated fatty acid oxidation promotes cell proliferation via nucleoside metabolism in nasopharyngeal carcinoma. Cell Death Dis.

[54] Camarda R, Zhou AY, Kohnz RA, Balakrishnan S, Mahieu C, Anderton B (2016). Inhibition of fatty acid oxidation as a therapy for MYC-overexpressing triple-negative breast cancer. Nat Med.

[55] Quan J, Li N, Tan Y, Liu H, Liao W, Cao Y (2022). PGC1alpha-mediated fatty acid oxidation promotes TGFbeta1-induced epithelial-mesenchymal transition and metastasis of nasopharyngeal carcinoma. Life Sci.

[56] Yan X, Zhang G, Bie F, Lv Y, Ma Y, Ma M (2017). Eugenol inhibits oxidative phosphorylation and fatty acid oxidation via downregulation of c-Myc/PGC-1beta/ERRalpha signaling pathway in MCF10A-ras cells. Sci Rep.

[57] Wang T, Fahrmann JF, Lee H, Li YJ, Tripathi SC, Yue C (2018). JAK/STAT3-Regulated fatty acid beta-oxidation is critical for breast Cancer Stem Cell Self-Renewal and Chemoresistance. Cell Metab.

[58] Li D, Li Y (2020). The interaction between ferroptosis and lipid metabolism in cancer. Signal Transduct Target Ther.

[59] Yang M, Liu K, Chen P, Zhu H, Wang J, Huang J (2022). Bromodomain-containing protein 4 (BRD4) as an epigenetic regulator of fatty acid metabolism genes and ferroptosis. Cell Death Dis.

[60] Yuan X, Kang Y, Dong J, Li R, Ye J, Fan Y (2023). Self-triggered thermoelectric nanoheterojunction for cancer catalytic and immunotherapy. Nat Commun.

[61] Kang Y, Xu L, Dong J, Yuan X, Ye J, Fan Y (2024). Programmed microalgae-gel promotes chronic wound healing in diabetes. Nat Commun.

[62] Aghebati-Maleki A, Dolati S, Ahmadi M, Baghbanzhadeh A, Asadi M, Fotouhi A (2020). Nanoparticles and cancer therapy: perspectives for application of nanoparticles in the treatment of cancers. J Cell Physiol.

[63] Barry NP, Sadler PJ (2013). Challenges for metals in medicine: how nanotechnology may help to shape the future. ACS Nano.

[64] Liu Y, Bhattarai P, Dai Z, Chen X (2019). Photothermal therapy and photoacoustic imaging via nanotheranostics in fighting cancer. Chem Soc Rev.

[65] Bockamp E, Rosigkeit S, Siegl D, Schuppan D. Nano-enhanced Cancer Immunotherapy: Immunology Encounters Nanotechnology. Cells. 2020;9(9). 10.3390/cells9092102 PMC7565449 32942725

[66] Perez-Herrero E, Fernandez-Medarde A (2015). Advanced targeted therapies in cancer: drug nanocarriers, the future of chemotherapy. Eur J Pharm Biopharm.

[67] Samaridou E, Heyes J, Lutwyche P (2020). Lipid nanoparticles for nucleic acid delivery: current perspectives. Adv Drug Deliv Rev.

[68] Moradi Kashkooli F, Soltani M, Souri M (2020). Controlled anti-cancer drug release through advanced nano-drug delivery systems: static and dynamic targeting strategies. J Control Release.

[69] Mitchell MJ, Billingsley MM, Haley RM, Wechsler ME, Peppas NA, Langer R (2021). Engineering precision nanoparticles for drug delivery. Nat Rev Drug Discov.

[70] Chen L, Mao Z, Wang Y, Kang Y, Wang Y, Mei L (2022). Edge modification facilitated heterogenization and exfoliation of two-dimensional nanomaterials for cancer catalytic therapy. Sci Adv.

[71] Huang L, Zhao S, Fang F, Xu T, Lan M, Zhang J (2021). Advances and perspectives in carrier-free nanodrugs for cancer chemo-monotherapy and combination therapy. Biomaterials.

[72] van der Meel R, Sulheim E, Shi Y, Kiessling F, Mulder WJM, Lammers T (2019). Smart cancer nanomedicine. Nat Nanotechnol.

[73] Hou YC, Zhang C, Zhang ZJ, Xia L, Rao KQ, Gu LH (2022). Aggregation-Induced Emission (AIE) and magnetic resonance imaging characteristics for targeted and image-guided siRNA therapy of Hepatocellular Carcinoma. Adv Healthc Mater.

[74] Jain P, Kathuria H, Momin M (2021). Clinical therapies and nano drug delivery systems for urinary bladder cancer. Pharmacol Ther.

[75] Xu M, Li S (2023). Nano-drug delivery system targeting tumor microenvironment: a prospective strategy for melanoma treatment. Cancer Lett.

[76] Xu C, Nam J, Hong H, Xu Y, Moon JJ (2019). Positron Emission Tomography-guided photodynamic therapy with biodegradable mesoporous silica nanoparticles for Personalized Cancer Immunotherapy. ACS Nano.

[77] Zhang Q, Kuang G, Wang H, Zhao Y, Wei J, Shang L (2023). Multi-bioinspired MOF Delivery systems from Microfluidics for Tumor Multimodal Therapy. Adv Sci (Weinh).

[78] Estape Senti M, Garcia Del Valle L, Schiffelers RM (2024). mRNA delivery systems for cancer immunotherapy: lipid nanoparticles and beyond. Adv Drug Deliv Rev.

[79] Qiu M, Tang Y, Chen J, Muriph R, Ye Z, Huang C et al. Lung-selective mRNA delivery of synthetic lipid nanoparticles for the treatment of pulmonary lymphangioleiomyomatosis. Proc Natl Acad Sci U S A. 2022;119(8). 10.1073/pnas.2116271119 PMC8872770 35173043

[80] Zhao L, Gu C, Gan Y, Shao L, Chen H, Zhu H (2020). Exosome-mediated siRNA delivery to suppress postoperative breast cancer metastasis. J Control Release.

[81] AlQahtani SA, Harisa GI, Alomrani AH, Alanazi FK, Badran MM (2021). Improved pharmacokinetic and biodistribution of 5-fluorouracil loaded biomimetic nanoerythrocytes decorated nanocarriers for liver cancer treatment. Colloids Surf B Biointerfaces.

[82] Wei G, Wang Y, Yang G, Wang Y, Ju R (2021). Recent progress in nanomedicine for enhanced cancer chemotherapy. Theranostics.

[83] Xu S, Cui K, Long K, Li J, Fan N, Lam WC (2023). Red Light-triggered anti-angiogenic and photodynamic combination therapy of age-related Macular Degeneration. Adv Sci (Weinh).

[84] Alamzadeh Z, Beik J, Pirhajati Mahabadi V, Abbasian Ardekani A, Ghader A, Kamrava SK (2019). Ultrastructural and optical characteristics of cancer cells treated by a nanotechnology based chemo-photothermal therapy method. J Photochem Photobiol B.

[85] Li J, Wang Q, Xia G, Adilijiang N, Li Y, Hou Z et al. Recent advances in targeted drug delivery strategy for enhancing Oncotherapy. Pharmaceutics. 2023;15(9). 10.3390/pharmaceutics15092233 PMC10534854 37765202

[86] Vilimas T, Wang AQ, Patnaik S, Hughes EA, Singleton MD, Knotts Z (2018). Pharmacokinetic evaluation of the PNC disassembler metarrestin in wild-type and Pdx1-Cre;LSL-Kras(G12D/+);Tp53(R172H/+) (KPC) mice, a genetically engineered model of pancreatic cancer. Cancer Chemother Pharmacol.

[87] Liu M, Peng Y, Nie Y, Liu P, Hu S, Ding J (2020). Co-delivery of doxorubicin and DNAzyme using ZnO@polydopamine core-shell nanocomposites for chemo/gene/photothermal therapy. Acta Biomater.

[88] Di Nottia M, Verrigni D, Torraco A, Rizza T, Bertini E, Carrozzo R. Mitochondrial dynamics: Molecular mechanisms, related primary mitochondrial disorders and therapeutic approaches. Genes (Basel). 2021;12(2). 10.3390/genes12020247 PMC7916359 33578638

[89] Li C, Zhang W, Liu S, Hu X, Xie Z (2020). Mitochondria-Targeting Organic nanoparticles for enhanced Photodynamic/Photothermal therapy. ACS Appl Mater Interfaces.

[90] Tan Y, Zhu Y, Zhao Y, Wen L, Meng T, Liu X (2018). Mitochondrial alkaline pH-responsive drug release mediated by Celastrol loaded glycolipid-like micelles for cancer therapy. Biomaterials.

[91] Liu Y, Zhou Z, Hou J, Xiong W, Kim H, Chen J (2022). Tumor Selective metabolic reprogramming as a prospective PD-L1 Depression Strategy to Reactivate Immunotherapy. Adv Mater.

[92] Zhang L, Wang D, Yang K, Sheng D, Tan B, Wang Z (2018). Mitochondria-targeted Artificial Nano-RBCs for amplified synergistic Cancer phototherapy by a single NIR Irradiation. Adv Sci (Weinh).

[93] Jiang L, Li L, He X, Yi Q, He B, Cao J (2015). Overcoming drug-resistant lung cancer by paclitaxel loaded dual-functional liposomes with mitochondria targeting and pH-response. Biomaterials.

[94] Zhu XJ, Li RF, Xu L, Yin H, Chen L, Yuan Y (2019). A novel self-assembled Mitochondria-Targeting protein nanoparticle acting as Theranostic platform for Cancer. Small.

[95] Kim HR, Cho HB, Lee S, Park JI, Kim HJ, Park KH (2023). Fusogenic liposomes encapsulating mitochondria as a promising delivery system for osteoarthritis therapy. Biomaterials.

[96] Cao S, Xia Y, Shao J, Guo B, Dong Y, Pijpers IAB (2021). Biodegradable polymersomes with structure inherent fluorescence and targeting capacity for enhanced photo-dynamic therapy. Angew Chem Int Ed Engl.

[97] Bao W, Liu M, Meng J, Liu S, Wang S, Jia R (2021). MOFs-based nanoagent enables dual mitochondrial damage in synergistic antitumor therapy via oxidative stress and calcium overload. Nat Commun.

[98] Yoong SL, Wong BS, Zhou QL, Chin CF, Li J, Venkatesan T (2014). Enhanced cytotoxicity to cancer cells by mitochondria-targeting MWCNTs containing platinum(IV) prodrug of cisplatin. Biomaterials.

[99] Yang X, Wang D, Zhu J, Xue L, Ou C, Wang W (2019). Functional black phosphorus nanosheets for mitochondria-targeting photothermal/photodynamic synergistic cancer therapy. Chem Sci.

[100] Lin LS, Wang JF, Song J, Liu Y, Zhu G, Dai Y (2019). Cooperation of endogenous and exogenous reactive oxygen species induced by zinc peroxide nanoparticles to enhance oxidative stress-based cancer therapy. Theranostics.

[101] Hsieh CH, Hsieh HC, Shih FS, Wang PW, Yang LX, Shieh DB (2021). An innovative NRF2 nano-modulator induces lung cancer ferroptosis and elicits an immunostimulatory tumor microenvironment. Theranostics.

[102] Sun Z, Chen W, Liu J, Yu B, Jiang C, Lu L (2021). Mitochondria-Targeting enhanced phototherapy by intrinsic characteristics Engineered one-for-all nanoparticles. ACS Appl Mater Interfaces.

[103] Sun L, Liu Y, Yang N, Ye X, Liu Z, Wu J (2023). Gold nanoparticles inhibit tumor growth via targeting the Warburg effect in a c-Myc-dependent way. Acta Biomater.

[104] Hernandes EP, Lazarin-Bidoia D, Bini RD, Nakamura CV, Cotica LF, de Oliveira Silva Lautenschlager S. Doxorubicin-loaded Iron oxide nanoparticles induce oxidative stress and cell cycle arrest in breast Cancer cells. Antioxid (Basel). 2023;12(2). 10.3390/antiox12020237 PMC9952039 36829796

[105] Jahanbani J, Ghotbi M, Shahsavari F, Seydi E, Rahimi S, Pourahmad J (2020). Selective anticancer activity of superparamagnetic iron oxide nanoparticles (SPIONs) against oral tongue cancer using in vitro methods: the key role of oxidative stress on cancerous mitochondria. J Biochem Mol Toxicol.

[106] Jiang F, Lee C, Zhang W, Jiang W, Cao Z, Chong HB (2022). Radiodynamic therapy with CsI(na)@MgO nanoparticles and 5-aminolevulinic acid. J Nanobiotechnol.

[107] Li Z, Guo D, Yin X, Ding S, Shen M, Zhang R (2020). Zinc oxide nanoparticles induce human multiple myeloma cell death via reactive oxygen species and Cyt-C/Apaf-1/Caspase-9/Caspase-3 signaling pathway in vitro. Biomed Pharmacother.

[108] Singh N, Kim J, Kim J, Lee K, Zunbul Z, Lee I (2023). Covalent organic framework nanomedicines: Biocompatibility for advanced nanocarriers and cancer theranostics applications. Bioact Mater.

[109] Zhang Y, Jia Q, Nan F, Wang J, Liang K, Li J (2023). Carbon dots nanophotosensitizers with tunable reactive oxygen species generation for mitochondrion-targeted type I/II photodynamic therapy. Biomaterials.

[110] Zuo L, Nie W, Yu S, Zhuang WR, Liang C, Li S (2023). Biomimetic nanovesicle with Mitochondria-Synthesized Sonosensitizer and Mitophagy Inhibition for Cancer Sono-Immunotherapy. Nano Lett.

[111] Dai W, Deng Y, Chen X, Huang Y, Hu H, Jin Q (2022). A mitochondria-targeted supramolecular nanoplatform for peroxynitrite-potentiated oxidative therapy of orthotopic hepatoma. Biomaterials.

[112] Zha S, Liu H, Li H, Li H, Wong KL, All AH (2024). Functionalized nanomaterials capable of crossing the blood-brain barrier. ACS Nano.

[113] Xiong H, Ye J, Wang M, Wang Y, Liu X, Jiang H (2022). In-situ bio-assembled specific au NCs-Aptamer-pyro conjugates nanoprobe for tumor imaging and mitochondria-targeted photodynamic therapy. Biosens Bioelectron.

[114] Zhang J, Yin X, Li C, Yin X, Xue Q, Ding L (2022). A multifunctional Photoacoustic/Fluorescence Dual-Mode-Imaging Gold-based Theranostic Nanoformulation without External Laser limitations. Adv Mater.

[115] Gong N, Ma X, Ye X, Zhou Q, Chen X, Tan X (2019). Carbon-dot-supported atomically dispersed gold as a mitochondrial oxidative stress amplifier for cancer treatment. Nat Nanotechnol.

[116] Liu B, Bian Y, Liang S, Yuan M, Dong S, He F (2022). One-step integration of Tumor Microenvironment-Responsive Calcium and Copper Peroxides Nanocomposite for enhanced Chemodynamic/Ion-Interference therapy. ACS Nano.

[117] Xu S, Zhang P, Heing-Becker I, Zhang J, Tang P, Bej R (2022). Dual tumor- and subcellular-targeted photodynamic therapy using glucose-functionalized MoS(2) nanoflakes for multidrug-resistant tumor ablation. Biomaterials.

[118] Qi J, Xiong Y, Cheng K, Huang Q, Cao J, He F (2021). Heterobifunctional PEG-grafted black phosphorus quantum dots: three-in-one nano-platforms for mitochondria-targeted photothermal cancer therapy. Asian J Pharm Sci.

[119] Zhou Z, Liu Y, Song W, Jiang X, Deng Z, Xiong W (2022). Metabolic reprogramming mediated PD-L1 depression and hypoxia reversion to reactivate tumor therapy. J Control Release.

[120] Mittal L, Camarillo IG, Varadarajan GS, Srinivasan H, Aryal UK, Sundararajan R (2020). High-throughput, label-free quantitative proteomic studies of the Anticancer effects of Electrical pulses with Turmeric Silver nanoparticles: an in vitro Model Study. Sci Rep.

[121] Zhou Z, Vazquez-Gonzalez M, Willner I (2021). Stimuli-responsive metal-organic framework nanoparticles for controlled drug delivery and medical applications. Chem Soc Rev.

[122] Lv C, Kang W, Liu S, Yang P, Nishina Y, Ge S (2022). Growth of ZIF-8 nanoparticles in situ on Graphene Oxide nanosheets: a multifunctional nanoplatform for combined Ion-Interference and Photothermal Therapy. ACS Nano.

[123] Yang F, Yu W, Yu Q, Liu X, Liu C, Lu C (2023). Mitochondria-targeted nanosystem with reactive oxygen species-controlled release of CO to enhance photodynamic therapy of PCN-224 by Sensitizing Ferroptosis. Small.

[124] Wu J, Ning P, Gao R, Feng Q, Shen Y, Zhang Y (2020). Programmable ROS-Mediated Cancer Therapy via Magneto-inductions. Adv Sci (Weinh).

[125] Liu T, Xiong CF, Zhang LJ, Jiao GH, Shi H, Feng J (2023). Boosting Doxorubicin-Induced Mitochondria apoptosis for the monodrug-mediated combination of Chemotherapy and Chemodynamic Therapy. Adv Healthc Mater.

[126] Cheng Y, Ji Y, Tong J (2020). Triple stimuli-responsive supramolecular nanoassembly with mitochondrial targetability for chemophotothermal therapy. J Control Release.

[127] Wu S, Xu L, He C, Wang P, Qin J, Guo F (2023). Lactate Efflux inhibition by Syrosingopine/LOD co-loaded Nanozyme for Synergetic Self-Replenishing Catalytic Cancer Therapy and Immune Microenvironment Remodeling. Adv Sci (Weinh).

[128] Hu H, Deng X, Song Q, Yang W, Zhang Y, Liu W (2021). Mitochondria-targeted accumulation of oxygen-irrelevant free radicals for enhanced synergistic low-temperature photothermal and thermodynamic therapy. J Nanobiotechnol.

[129] Truong Hoang Q, Huynh KA, Nguyen Cao TG, Kang JH, Dang XN, Ravichandran V (2023). Piezocatalytic 2D WS(2) nanosheets for Ultrasound-Triggered and Mitochondria-targeted Piezodynamic Cancer Therapy Synergized with Energy metabolism-targeted chemotherapy. Adv Mater.

[130] Zhou Z, Zheng C, Liu Y, Luo W, Deng H, Shen J (2022). Chitosan biguanide induced mitochondrial inhibition to amplify the efficacy of oxygen-sensitive tumor therapies. Carbohydr Polym.

[131] Yang Z, Wang J, Liu S, Li X, Miao L, Yang B (2020). Defeating relapsed and refractory malignancies through a nano-enabled mitochondria-mediated respiratory inhibition and damage pathway. Biomaterials.

[132] Zhang J, Yan L, Wei P, Zhou R, Hua C, Xiao M (2021). PEG-GO@XN nanocomposite suppresses breast cancer metastasis via inhibition of mitochondrial oxidative phosphorylation and blockade of epithelial-to-mesenchymal transition. Eur J Pharmacol.

[133] Guo W, Chen Z, Feng X, Shen G, Huang H, Liang Y (2021). Graphene oxide (GO)-based nanosheets with combined chemo/photothermal/photodynamic therapy to overcome gastric cancer (GC) paclitaxel resistance by reducing mitochondria-derived adenosine-triphosphate (ATP). J Nanobiotechnol.

[134] Hu XK, Rao SS, Tan YJ, Yin H, Luo MJ, Wang ZX (2020). Fructose-coated Angstrom silver inhibits osteosarcoma growth and metastasis via promoting ROS-dependent apoptosis through the alteration of glucose metabolism by inhibiting PDK. Theranostics.

[135] Bhattacharya SR, Bhattacharya K, Xavier VJ, Ziarati A, Picard D, Burgi T (2022). The Atomically Precise Gold/Captopril nanocluster au(25)(Capt)(18) gains anticancer activity by inhibiting mitochondrial oxidative phosphorylation. ACS Appl Mater Interfaces.

[136] Zhou W, Chen S, Ouyang Y, Huang B, Zhang H, Zhang W (2023). A supramolecular nanoplatform for imaging-guided phototherapies via hypoxia tumour microenvironment remodeling. Chem Sci.

[137] Lu L, Liu G, Lin C, Li K, He T, Zhang J (2021). Mitochondrial metabolism targeted nanoplatform for efficient triple-negative breast Cancer Combination Therapy. Adv Healthc Mater.

[138] Zou Y, Sun Y, Wang Y, Zhang D, Yang H, Wang X (2023). Cancer cell-mitochondria hybrid membrane coated Gboxin loaded nanomedicines for glioblastoma treatment. Nat Commun.

[139] Zhang K, Zhu J, Wang R, Zhu W, Zhang Z, Gong L (2023). Mitochondria-anchoring self-assembled nanoparticles for multi-path energy depletion: a nano bomb in chemo-co-starvation therapy. Int J Pharm.

[140] Yu X, Lyu M, Ou X, Liu W, Yang X, Ma X (2023). AIEgens/Mitochondria Nanohybrids as Bioactive Microwave Sensitizers for Non-thermal Microwave Cancer Therapy. Adv Healthc Mater.

[141] Liu J, Liu X, Wu M, Qi G, Liu B (2020). Engineering Living Mitochondria with AIE Photosensitizer for Synergistic Cancer cell ablation. Nano Lett.

[142] Zeng WN, Yu QP, Wang D, Liu JL, Yang QJ, Zhou ZK (2021). Mitochondria-targeting graphene oxide nanocomposites for fluorescence imaging-guided synergistic phototherapy of drug-resistant osteosarcoma. J Nanobiotechnol.

[143] Zhang H, Liu R, Wan P, You X, Li S, Liu Z (2023). Targeting tumor energy metabolism via simultaneous inhibition of mitochondrial respiration and glycolysis using biodegradable hydroxyapatite nanorods. Colloids Surf B Biointerfaces.

[144] Ding XL, Liu MD, Cheng Q, Guo WH, Niu MT, Huang QX (2022). Multifunctional liquid metal-based nanoparticles with glycolysis and mitochondrial metabolism inhibition for tumor photothermal therapy. Biomaterials.

[145] Le XT, Lee J, Nguyen NT, Lee WT, Lee ES, Oh KT (2022). Combined phototherapy with metabolic reprogramming-targeted albumin nanoparticles for treating breast cancer. Biomater Sci.

[146] Wang H, Shi W, Zeng D, Huang Q, Xie J, Wen H (2021). pH-activated, mitochondria-targeted, and redox-responsive delivery of paclitaxel nanomicelles to overcome drug resistance and suppress metastasis in lung cancer. J Nanobiotechnol.

[147] Li F, Liu Y, Dong Y, Chu Y, Song N, Yang D (2022). Dynamic assembly of DNA nanostructures in living cells for mitochondrial interference. J Am Chem Soc.

[148] Dahabiyeh LA, Mahmoud NN, Al-Natour MA, Safo L, Kim DH, Khalil EA et al. Phospholipid-gold nanorods induce Energy Crisis in MCF-7 cells: cytotoxicity evaluation using LC-MS-Based Metabolomics Approach. Biomolecules. 2021;11(3). 10.3390/biom11030364 PMC7997200 33673519

[149] Wang YY, Zhang XY, Li SL, Jiang FL, Jiang P, Liu Y (2023). AuPt-Loaded Cu-Doped polydopamine nanocomposites with multienzyme-mimic activities for dual-modal imaging-guided and cuproptosis-enhanced Photothermal/Nanocatalytic therapy. Anal Chem.

[150] Jiang Y, Tan Y, Xiao K, Li X, Shao K, Song J (2021). pH-Regulating nanoplatform for the Double Channel Chase of Tumor Cells by the Synergistic Cascade between Chlorine Treatment and Methionine-Depletion Starvation Therapy. ACS Appl Mater Interfaces.

[151] She W, Li H, Wang Z, Liu T, Zhao D, Guo Z (2024). Site-specific controlled-release nanoparticles for immune reprogramming via dual metabolic inhibition against triple-negative breast cancer. J Control Release.

[152] Nguyen Cao TG, Truong Hoang Q, Kang JH, Kang SJ, Ravichandran V, Rhee WJ (2023). Bioreducible exosomes encapsulating glycolysis inhibitors potentiate mitochondria-targeted sonodynamic cancer therapy via cancer-targeted drug release and cellular energy depletion. Biomaterials.

[153] Yang R, Fang XL, Zhen Q, Chen QY, Feng C (2019). Mitochondrial targeting nano-curcumin for attenuation on PKM2 and FASN. Colloids Surf B Biointerfaces.

[154] Wang J, Zhang L, Xin H, Guo Y, Zhu B, Su L (2022). Mitochondria-targeting folic acid-modified nanoplatform based on mesoporous carbon and a bioactive peptide for improved colorectal cancer treatment. Acta Biomater.

[155] Liu X, Li Y, Wang K, Chen Y, Shi M, Zhang X (2021). GSH-Responsive Nanoprodrug to inhibit glycolysis and alleviate immunosuppression for Cancer Therapy. Nano Lett.

[156] Yang T, Zhang X, Yang X, Li Y, Xiang J, Xiang C (2023). A mitochondria-targeting self-assembled carrier-free lonidamine nanodrug for redox-activated drug release to enhance cancer chemotherapy. J Mater Chem B.

[157] Machuca A, Garcia-Calvo E, Anunciacao DS, Luque-Garcia JL. Integration of Transcriptomics and Metabolomics to reveal the Molecular mechanisms Underlying Rhodium nanoparticles-based photodynamic Cancer therapy. Pharmaceutics. 2021;13(10). 10.3390/pharmaceutics13101629 PMC8539937 34683922

[158] Zhang Y, Ren Y, Xu H, Li L, Qian F, Wang L (2023). Cascade-Responsive 2-DG Nanocapsules encapsulate aV-siCPT1C conjugates to inhibit Glioblastoma through multiple inhibition of Energy Metabolism. ACS Appl Mater Interfaces.

[159] Gao Y, Song Z, Jia L, Tang Y, Wang C, Zhao X (2022). Self-amplified ROS production from fatty acid oxidation enhanced tumor immunotherapy by atorvastatin/PD-L1 siRNA lipopeptide nanoplexes. Biomaterials.

[160] Wang H, Lin M, Chen G, Xiao Z, Shuai X (2023). Nanodrug regulates ROS homeostasis via enhancing fatty acid oxidation and inhibiting autophagy to overcome tumor drug resistance. Biomater Sci.

[161] Luo L, Li X, Zhang J, Zhu C, Jiang M, Luo Z (2021). Enhanced immune memory through a constant photothermal-metabolism regulation for cancer prevention and treatment. Biomaterials.

[162] Wise DR, Thompson CB (2010). Glutamine addiction: a new therapeutic target in cancer. Trends Biochem Sci.

[163] Mullen AR, Wheaton WW, Jin ES, Chen PH, Sullivan LB, Cheng T (2011). Reductive carboxylation supports growth in tumour cells with defective mitochondria. Nature.

[164] Tang E, Liu S, Zhang Z, Zhang R, Huang D, Gao T (2021). Therapeutic potential of glutamine pathway in Lung Cancer. Front Oncol.

[165] Ren J, Zhou J, Liu H, Jiao X, Cao Y, Xu Z (2021). Ultrasound (US)-activated redox dyshomeostasis therapy reinforced by immunogenic cell death (ICD) through a mitochondrial targeting liposomal nanosystem. Theranostics.

[166] Wang Q, Li S, Xu C, Hua A, Wang C, Xiong Y (2023). A novel lonidamine derivative targeting mitochondria to eliminate cancer stem cells by blocking glutamine metabolism. Pharmacol Res.

[167] Garbincius JF, Elrod JW (2022). Mitochondrial calcium exchange in physiology and disease. Physiol Rev.

[168] Giorgi C, Marchi S, Pinton P (2018). The machineries, regulation and cellular functions of mitochondrial calcium. Nat Rev Mol Cell Biol.

[169] Zheng P, Ding B, Shi R, Jiang Z, Xu W, Li G (2021). A multichannel ca(2+) Nanomodulator for Multilevel mitochondrial Destruction-mediated Cancer Therapy. Adv Mater.

[170] Shao F, Han J, Tian Z, Wang Z, Liu S, Wu Y (2023). Synergistic ROS generation and directional overloading of endogenous calcium induce mitochondrial dysfunction in living cells. Biomaterials.

[171] Wang X, Li Y, Deng X, Jia F, Cui X, Lu J (2021). Colloidally stabilized DSPE-PEG-Glucose/Calcium phosphate hybrid nanocomposites for enhanced photodynamic Cancer Therapy via complementary mitochondrial ca(2+) overload and autophagy inhibition. ACS Appl Mater Interfaces.

[172] Ahmad A, Rashid S, Chaudhary AA, Alawam AS, Alghonaim MI, Raza SS (2023). Nanomedicine as potential cancer therapy via targeting dysregulated transcription factors. Semin Cancer Biol.

[173] Kang Y, Mao Z, Wang Y, Pan C, Ou M, Zhang H (2022). Design of a two-dimensional interplanar heterojunction for catalytic cancer therapy. Nat Commun.

[174] Gomes A, Sengupta J, Datta P, Ghosh S, Gomes A (2016). Physiological interactions of nanoparticles in Energy Metabolism, Immune function and their Biosafety: a review. J Nanosci Nanotechnol.

[175] Saraiva C, Praca C, Ferreira R, Santos T, Ferreira L, Bernardino L (2016). Nanoparticle-mediated brain drug delivery: overcoming blood-brain barrier to treat neurodegenerative diseases. J Control Release.

[176] Blanco E, Shen H, Ferrari M (2015). Principles of nanoparticle design for overcoming biological barriers to drug delivery. Nat Biotechnol.

[177] Gonzalez-Valdivieso J, Girotti A, Schneider J, Arias FJ (2021). Advanced nanomedicine and cancer: challenges and opportunities in clinical translation. Int J Pharm.

[178] Jin X, Yang H, Mao Z, Wang B (2021). Cathepsin B-responsive multifunctional peptide conjugated gold nanorods for mitochondrial targeting and precise photothermal cancer therapy. J Colloid Interface Sci.

[179] Yang GG, Pan ZY, Zhang DY, Cao Q, Ji LN, Mao ZW (2020). Precisely assembled nanoparticles against Cisplatin Resistance via Cancer-Specific Targeting of Mitochondria and Imaging-guided chemo-photothermal therapy. ACS Appl Mater Interfaces.

[180] Xiao Y, Zhang T, Ma X, Yang QC, Yang LL, Yang SC (2021). Microenvironment-responsive Prodrug-Induced pyroptosis boosts Cancer Immunotherapy. Adv Sci (Weinh).

[181] Shen R, Jiang Q, Li P, Wang D, Yu C, Meng T (2023). Targeted plus controlled - composite nano delivery system opens the tumor vascular and microenvironment normalization window for anti-tumor therapy. Int J Pharm.

[182] Kang X, Bu F, Feng W, Liu F, Yang X, Li H (2022). Dual-Cascade Responsive nanoparticles enhance pancreatic Cancer Therapy by eliminating tumor-resident intracellular Bacteria. Adv Mater.

[183] Elgogary A, Xu Q, Poore B, Alt J, Zimmermann SC, Zhao L (2016). Combination therapy with BPTES nanoparticles and metformin targets the metabolic heterogeneity of pancreatic cancer. Proc Natl Acad Sci U S A.

[184] Schlaepfer IR, Rider L, Rodrigues LU, Gijon MA, Pac CT, Romero L (2014). Lipid catabolism via CPT1 as a therapeutic target for prostate cancer. Mol Cancer Ther.

[185] Missiroli S, Perrone M, Genovese I, Pinton P, Giorgi C (2020). Cancer metabolism and mitochondria: finding novel mechanisms to fight tumours. EBioMedicine.

[186] Delaunay S, Pascual G, Feng B, Klann K, Behm M, Hotz-Wagenblatt A (2022). Mitochondrial RNA modifications shape metabolic plasticity in metastasis. Nature.

[187] Liu Y, Chen C, Wang X, Sun Y, Zhang J, Chen J (2022). An epigenetic role of Mitochondria in Cancer. Cells.

[188] Izzo V, Bravo-San Pedro JM, Sica V, Kroemer G, Galluzzi L (2016). Mitochondrial permeability transition: New findings and persisting uncertainties. Trends Cell Biol.

[189] Bonora M, Wieckowsk MR, Chinopoulos C, Kepp O, Kroemer G, Galluzzi L (2015). Molecular mechanisms of cell death: central implication of ATP synthase in mitochondrial permeability transition. Oncogene.

[190] Szabo L, Cummins N, Paganetti P, Odermatt A, Papassotiropoulos A, Karch C (2023). ER-mitochondria contacts and cholesterol metabolism are disrupted by disease-associated tau protein. EMBO Rep.

[191] Fransen M, Lismont C, Walton P. The peroxisome-mitochondria connection: how and why? Int J Mol Sci. 2017;18(6). 10.3390/ijms18061126 PMC5485950 28538669

[192] de Montes P (2021). Mitochondria-plasma membrane interactions and communication. J Biol Chem.

[193] Hua S, de Matos MBC, Metselaar JM, Storm G (2018). Current trends and challenges in the clinical translation of Nanoparticulate nanomedicines: pathways for Translational Development and Commercialization. Front Pharmacol.

[194] Wen R, Banik B, Pathak RK, Kumar A, Kolishetti N, Dhar S (2016). Nanotechnology inspired tools for mitochondrial dysfunction related diseases. Adv Drug Deliv Rev.

